# Surfaceome analyses uncover CD98hc as an antibody drug-conjugate target in triple negative breast cancer

**DOI:** 10.1186/s13046-022-02330-4

**Published:** 2022-03-22

**Authors:** Juan Carlos Montero, Elisa Calvo-Jiménez, Sofía del Carmen, Mar Abad, Alberto Ocaña, Atanasio Pandiella

**Affiliations:** 1 Institute of Biomedical Research of Salamanca (IBSAL), Instituto de Biología Molecular y Celular del Cáncer (CSIC-Universidad de Salamanca) and CIBERONC, Salamanca, Spain; 2 Department of Pathology and IBSAL, University Hospital of Salamanca, University of Salamanca, 37007 Salamanca, Spain; 3 Symphogen, Copenhagen, Denmark

**Keywords:** CD98hc, Antibody-drug conjugates, Triple negative breast cancer, Targeted therapy

## Abstract

**Background:**

Despite the incorporation of novel therapeutics, advanced triple negative breast cancer (TNBC) still represents a relevant clinical problem. Considering this, as well as the clinical efficacy of antibody-drug conjugates (ADCs), we aimed at identifying novel ADC targets that could be used to treat TNBC.

**Methods:**

Transcriptomic analyses were performed on TNBC and normal samples from three different studies. Plasma membrane proteins of three cell lines representative of the TNBC subtype were identified by cell surface biotinylation or plasma membrane isolation, followed by analyses of cell surface proteins using the Surfaceome online tool. Immunofluorescence and western studies were used to characterize the action of a CD98hc-directed ADC, which was prepared by in house coupling of emtansine to an antibody that recognized the ectodomain of CD98hc. Xenografted TNBC cells were used to analyze the antitumoral properties of the anti-CD98hc ADC.

**Results:**

Comparative genomic studies between normal breast and TNBC tissues, together with proteomic and bioinformatic analyses resulted in the elaboration of a catalog of potential ADC targets. One of them, the CD98hc transmembrane protein, was validated as an ADC target. An antibody recognizing the ectodomain of CD98hc efficiently internalized and reached the lysosomal compartment. An emtansine-based ADC derived from such antibody was prepared and showed antitumoral properties in TNBC in vitro and in vivo models. Mechanistically, the anti-CD98hc ADC blocked cell cycle progression, that was followed by cell death caused by mitotic catastrophe.

**Conclusions:**

This work describes a list of potential ADC targets in TNBC and validates one of them, the transmembrane protein CD98hc. The studies presented here also demonstrate the robustness of the multiomic approach herewith described to identify novel potential ADC targets.

**Supplementary Information:**

The online version contains supplementary material available at 10.1186/s13046-022-02330-4.

## Background

Breast cancer is the most frequently diagnosed cancer in women [1, 2]. It is a heterogeneous disease that is classified immunohistochemically into three main subtypes: estrogen or progesterone receptor positive tumors; those which overexpress the human epidermal growth factor receptor 2 (HER2); and triple negative breast cancers (TNBCs) [3]. The latter are characterized by the lack of hormonal receptors and absence of HER2 overexpression. They represent 15-20% of all breast tumors and usually display poorer prognosis than other subtypes [3, 4]. TNBCs are chemotherapy-sensitive and this treatment remains the standard of care, despite its limited benefit in advances stages [4, 5]. Because of such limited clinical benefit, extensive research is being carried out to discover potential new therapeutically actionable targets for this type of breast tumors [6]. These efforts recently led to the development of PARP inhibitors such as olaparib and talazoparib. In 2018, the FDA approved the use of those drugs to treat advanced-stage HER2-negative breast cancer in individuals with *BRCA1* or *BRCA2* mutation [7]. Another recently approved targeted therapy for TNBC is based on the use of immune checkpoint inhibitors. In fact, clinical studies demonstrated the efficacy of PD-L1 blockade by atezolizumab in combination with nanoparticle albumin-bound (nab)-paclitaxel for the treatment of patients with previously untreated metastatic TNBC [8, 9]. Yet, a limited number of patients responds to PARP or immune checkpoint inhibitors, and those who respond often develop resistance and relapse [3].

An additional therapeutic strategy that has reported clinical benefit in TNBC is the use of antibody-drug conjugates (ADCs). ADCs consist of an antibody backbone to which a potent cytotoxic agent is bound through a chemical linker [10]. The antibody recognizes a cell surface protein expressed in tumoral cells, facilitating the delivery of the cytotoxic to those target-expressing cells. At present, while several ADCs are currently being tested in the clinic for patients with metastatic TNBC, only one, sacituzumab govitecan, has been approved for the treatment of advanced metastatic TNBC [11–13]. Sacituzumab govitecan has been developed against trophoblast cell surface antigen 2 (TROP2), a transmembrane protein expressed in TNBC. The antitumoral efficacy of sacituzumab govitecan in TNBC and that of other ADCs in solid and hematological tumors open the fascinating possibility of identifying targets against which novel ADCs could be developed to fight TNBC.

We initiated a study to generate a catalog of cell surface proteins expressed in TNBC with the purpose of defining potential ADC targets for the therapy of that disease. Genomic and proteomic studies allowed the identification of several membrane proteins differentially expressed in TNBC versus normal non-tumoral breast cells. These studies led to the identification of CD98 as a differentially expressed protein complex in TNBC. CD98 is a heteromeric solute carrier formed by two subunits, a heavy and a light chain [14]. The heavy chain, known as CD98hc or SLC3A2, is an ≈80 kDa type II transmembrane protein which exposes most of its structure to the extracellular space. CD98hc is linked through disulfide bonds to the second component of the dimer, the light chain, which is one of the several ≈40 kDa multi-pass amino acid transporters, such as LAT1 or LAT2 [14–16]. The heteromeric CD98hc-LAT complex participates in amino acid uptake and its correct assembly is required for proper membrane targeting [14, 16]. An ADC against CD98hc was constructed by linking the potent antimicrotubular drug DM1 to an antibody selected to react with the ectodomain of human CD98hc. The anti-CD98hc ADC was efficiently internalized and reached the lysosomal compartment, where it was proteolytically processed delivering to the cytosol the potent antimicrotubular payload. In vitro and in vivo studies demonstrated a potent and specific antitumoral activity of this anti-CD98hc ADC against human TNBC cell lines and tumors. These studies uncover a new ADC target and open the possibility of exploring the activity of the anti-CD98hc ADC in the TNBC clinic.

## Methods

### Reagents and antibodies

Dulbecco’s modified Eagle medium (DMEM), fetal bovine serum (FBS), penicillin and streptomycin were purchased from Life Technologies (Carlsbad, CA, USA). Proteinase K, 6-diamidino-2-phenylindole (DAPI) and 3-(4, 5-Dimethylthiazol-2-yl)-2, 5-Diphenyltetrazolium Bromide (MTT) were from Sigma-Aldrich (St Louis, MO, USA). Immobilon®-P transfer membranes were from Merck Millipore Corp. (Darmstadt, Germany). The Safe & Easy Toxin (SET™) was from Levena Biopharma (San Diego, CA, USA). Other generic chemicals were from Sigma-Aldrich (St. Louis, MO, USA), USB Corporation (Cleveland, OH, USA) Roche Biochemicals (Hoffmann, Germany), or Merck (Darmstadt, Germany).

Antibodies against calnexin, β-actin, cyclin B1, CD98hc (used in: Immunofluorescence, cell surface immunoprecipitation, cell surface staining, cytometer and to prepare the ADC) and GAPDH were from Santa Cruz Biotechnology (Santa Cruz, CA, USA). Antibodies against CD98hc (used in Western blot experiments), LAT1, GLUT1, LAMP1, phospho-HA2X (S139), phospho-Rb (S780), phospho-Rb (S807/811), phospho-cdc2 (Y15), Wee1, PARP and cleaved caspase 3 were from Cell Signaling Technologies (Beverly, MA, USA). Antibodies against BUBR1 and nucleoporin were from BD transduction Laboratories (San Jose, CA, USA). The anti phospho-H3 (S10) was from Merck Millipore (Darmstadt, Germany). The anti β -tubulin was from Sigma. The anti-DM1 antibody prepared against BSA-coupled DM1 will be described elsewhere. Secondary HRP-conjugated antibodies recognizing mouse IgG and rabbit IgG were obtained from GE Healthcare Life Sciences (Piscataway, NJ, USA) and Bio-Rad Laboratories (Hercules, CA, USA), respectively.

### Cell culture, lentivirus production and infection

BT549, HCC70, HCC1187, HCC1937, HCC3153, HS578T, MDA-MB231 and MDA-MB468 cells were grown in Dulbecco's modified Eagle's medium (DMEM) or in RPMI 1640 medium supplemented with 10% fetal bovine serum (FBS), containing high glucose (4500 mg/liter) and antibiotics (penicillin 100 U/ml, streptomycin 100 µg/ml). Cell lines were cultured at 37°C in a humidified atmosphere in the presence of 5% CO_2_ and 95% air, as described [17].

For lentivirus production, 4 µg of the following plasmids: pMDLg/RRE, pRSV-Rev and pMD2.G (Addgene, Cambridge, MA, USA), along with 8 µg of the pLKO.1 lentiviral plasmid containing a scramble shRNA (sh-Control) or the shRNAs for CD98hc, GLUT1 or LAT1 (GE Dharmacon, Lafayette, CO, USA) were co-transfected into HEK293T cells [18] using JetPEI® reagent (Polyplus-transfection, Illkirch, France), following the manufacturer’s instructions. Twenty-four hours later, HEK293T medium was replaced with fresh medium and 48 hours after the co-transfection, the medium containing lentiviral particles was collected, filtered and used to infect BT549 and MDA-MB231 cells after the addition of 6 µg/ml polybrene (Sigma-Aldrich, St. Louis, MO, USA). The cells were cultured for 48 hours to allow for efficient protein knockdown and were subsequently selected with 3 µg/ml puromycin (Sigma-Aldrich, St. Louis, MO, USA) for another 48 hours. A minimum of 5 different shRNA sequences targeting CD98hc, GLUT1 or LAT1 were tested and those that produced higher knockdown were used (#3 and #7 for CD98hc, # 5 for GLUT1 and #9 y #12 for LAT1).

### Immunoprecipitation and Western blotting

The procedures for the preparation of cell extracts for protein analyses, immunoprecipitation and Western blotting have been described [19, 20]. GAPDH, β -actin or calnexin were used as loading controls. Densitometric measurements of the bands were performed using the Image Lab^TM^ Software Version 6.0.1 Bio-Rad Laboratories (Hercules, CA, USA), which was provided with a ChemiDoc apparatus. Stain free blot was performed by adding 50 microliters of 2,2,2-Trichloroethanol to 10 ml of the SDS-PAGE gel solution. Detection of total protein was made in the ChemiDoc apparatus (Bio-Rad) following the manufacturer's instructions.

### Immunofluorescence microscopy

The immunofluorescence protocol has been detailed in [21]. Briefly, cells were cultured on glass coverslips inserted into 35 mm dishes and treated with 10 nM anti-CD98hc or anti-CD98hc-DM1 for the indicated times. Then, cells on coverslips were washed with PBS/CM (1 mM CaCl_2_, 0.5 mM MgCl_2_ in PBS), fixed in 2% paraformaldehyde, and then washed with PBS/CM. Incubations were quenched with 50 mM NH_4_Cl. Cells were permeabilized (0.1% triton, 0.2% BSA) and then coverslips with cells incubated for 1 hour in blocking solution, that contained PBS/CM with 0.2% BSA. The anti-LAMP1 (dilution 1:200), anti- β -tubulin (dilution 1:200) or anti-nucleoporin (dilution 1:200) antibodies were added to the cells on the coverslips in blocking solution for 2 hours at room temperature. After three washes for 10 minutes each in PBS with 0.2 % BSA, the coverslips were incubated with Cy3-conjugated anti-mouse, Cy2-conjugated goat anti-rabbit or Cy2-conjugated anti-mouse antibodies in blocking solution for 30 minutes. Coverslips were washed three times for 10 minutes each in PBS with 0.2 % BSA, stained with DAPI and mounted. Samples were analyzed by confocal immunofluorescence microscopy using a Leica TCS SP5 system (Leica Microsystems CMS, Wetzlar, Germany).

### Xenograft studies

Mice were handled at the institute’s animal facility, and all treatments were done in accordance with the legal and institutional guidelines. Female BALB/c nude mice, 7 weeks old, were obtained from Charles River Laboratories (Wilmington, MA, USA). A total of 1x10^6^ MDA-MB231 cells in 50 µl of DMEM+10% fetal bovine serum and 50 µl of Matrigel (BD Biosciences) were injected into the mammary fat pad. When tumors reached 45 mm^3^, animals were randomized into 2 groups (*n*=6 animals for each condition, each bearing two tumors), and treatments were initiated by weekly intraperitoneal injection with anti-CD98hc-DM1 (first dose 6 mg/Kg, second and third doses 15 mg/Kg). Tumor diameters were serially measured with a digital caliper (Proinsa, Vitoria, Spain) every 3-4 days (twice a week), and tumor volumes were calculated using the following formula: V = (L × W^2^)/2, where V = volume (cubic millimeters), L = length (millimeters), and W = width (millimeters). Tumors samples were obtained on day 31 after initiation of treatments after killing the animals by CO_2_ inhalation, and immediately one piece was frozen in liquid nitrogen. Tumors that were frozen in liquid nitrogen were minced, washed with phosphate buffered saline, and homogenized (Dispomix, L&M Biotech, Holly Springs, NC, USA) in ice-cold lysis buffer (2 ml/100 mg of tumor). This homogenate was centrifuged at 10,000 x *g* for 20 min at 4°C, and the supernatants were transferred to new tubes. The levels of expression of different proteins and IgG-DM1 were analyzed by western.

### Protease protection experiments

BT549, HS578T, MDA-MB231 and HCC3153 cells were washed once with KRH buffer (that contained, in mmol/liter: NaCl, 140; KCl, 5; CaCl_2_, 2; MgSO_4_, 1.2; KH_2_PO_4_, 1.2; glucose, 6; Hepes, 25, pH 7.4) and incubated in this buffer supplemented with 200 µg/ml proteinase K for 30 min [22]. After washing three times with PBS containing 2 mM PMSF, cells were lysed in 1 ml of lysis buffer with protease inhibitors. Analyses of the effect of proteinase K on plasma membrane CD98hc were carried out by immunoprecipitation (500 µg) and western blot of CD98hc with anti-CD98hc antibodies.

### Preparation of plasma membrane-enriched microsomes

Enrichment of plasma membrane microsomes was carried out as described [23]. MDA-MB231, BT549 and HS578T cells from ten 100-mm dishes (90% confluent) were washed with PBS and detached by incubation for 10 min at 37°C with 0.25 M sucrose, 1 mM EDTA, 1 mM PMSF, and 10 mM Tris, pH 7.0. The cell suspension was homogenized on ice with a tight-fitting Dounce homogenizer (Kontes Glass Company, Vineland, NJ). This homogenate was centrifuged at 4,000 x *g* for 10 min, and the resulting pellet was rehomogenized and centrifuged at the same speed. The supernatants from these two centrifugation steps were pooled and centrifuged at 30,000 x *g* for 30 min. The resulting pellet was resuspended in homogenization buffer, layered on top of a 36% (w/w) sucrose solution, and centrifuged at 100,000 x *g* for 60 min. The fraction of membranous material at the 36% interface was collected, sedimented at 30,000 x *g* for 30 min, and resuspended in an 8M urea-2M thiourea solution. This fraction, which is enriched in plasma membrane proteins was analyzed by mass spectrometry, as described [18].

### Cell surface biotinylation

Cell surface biotinylation was performed as described [24]. MDA-MB231, BT549 and HS578T cells were washed twice with ice-cold PBS and then incubated with NHS-LC-biotin (0.25 mg/ml) for 30 minutes at 4°C. Parallel cultures of cells were also processed but without incubation with the biotinylation reagent. The data obtained with them was used as non-specific background. Cells were washed twice with PBS and then incubated with PBS containing 50 mM NH_4_Cl for 10 min at 4°C. Cell monolayers were washed twice with PBS, lysed, and the lysates (7.5 mg) were precipitated with streptavidin-agarose for 2h at 4°C. The precipitates were then washed by centrifugation at 1,600 x g for 5 minutes. Then the resin was washed three times with buffer A (1% w/v NP40, 0.5% w/v SDS in PBS), once with Buffer B (0.1% w/v NP40, 0.5M NaCl in PBS) y once with digestion buffer (0.25 mM AMB-ammonium bicarbonate). Samples were run for 1 cm in 10% gels that were stained with Coomassie blue. A 1 cm^2^ fragment of the gel, was obtained and analyzed by mass spectrometry at our center’s proteomic facility.

### Cell surface immunoprecipitation

Cells were washed twice with Krebs-Ringer-HEPES buffer and incubated with 10 nM anti-CD98hc in the same buffer for 2 hours at 4º C. Monolayers were washed twice with PBS and lysed using lysis buffer. Cell debris were removed by centrifugation and supernatants incubated for 60 minutes with protein A-Sepharose. Immunocomplexes were then washed and loaded in SDS-PAGE gels. CD98hc was detected by western with the anti-CD98hc antibody.

### Cell proliferation and cell cycle assays

Cell proliferation was assessed by MTT metabolization and cell cycle by propidium iodide staining as previously described [25].

### Generation of anti-CD98hc-DM1

For the preparation of the ADC targeting CD98hc we used an anti-CD98hc antibody commercially available (Santa Cruz Biotechnology Company, sc-59145), together with the payload coupling kit Safe & Easy Toxin (SET™) SET0101 SMCC-DM1 (Levena Biopharma, San Diego, CA, USA). Preparation of the ADC was carried out following the manufacturer's instructions.

### Transcriptomic studies

Three different sources of transcriptomic data were analyzed. Two of them, TBC (Tokushima Breast care Clinic study) and HBS (Harvard Breast SPORE blood and tissue repository), were publicly available [26, 27] and the third one, SUH (Salamanca University Hospital), resulted from analyses of patient samples (fifteen tumor and one normal sample) obtained at our University Hospital in Salamanca. Briefly, 10-20 mg of each sample was used to isolate total RNA using PureLink RNA Mini Kit (Ambion, Life Technologies, Carlsbad, CA, USA) according to the manufacturer’s instructions. The RNA integrity was assessed using Agilent 2100 Bioanalyzer (Agilent, Palo Alto, CA, USA). Labelling and hybridizations were performed according to protocols from Affymetrix. Briefly, 100 ng of total RNA were amplified and labeled using the WT Plus reagent kit (Affymetrix) and then hybridized to Human Gene 1.0 ST Array (Affymetrix). Washing and scanning were performed using GeneChip System of Affymetrix (GeneChip Hybridization Oven 645, GeneChip Fluidics Station 450 and GeneChip Scanner 7G). The HBS and TBC microarrays data were downloaded from the public National Center for Biotechnology Information (NCBI) GEO database (https://www.ncbi.nlm.nih.gov/gds), with accession no. GSE3744 and GSE38959, respectively [27]. The array data from the SUH was analyzed using the dChip software [28]. TBC and HBS array data were analyzed by Bioconductor from R software, using RMA function [29] for data normalization and Limma package [30] for differential expression identification based on an ANOVA test. The differentially expressed genes were selected using a *p* value of less than 0.05 and a log fold change of >1.5 as thresholds.

The data comparing SLC3A2 gene expression in normal and tumor tissue of breast cancer and in the different subtypes of breast cancer were obtained from TNMplot (https://tnmplot.com/analysis/) and UCSC Xena (https://xena.ucsc.edu/), respectively.

### Immunohistochemical analyses of CD98hc

TNBC samples were formalin fixed and paraffin embedded. Three-micrometer sections were cut with a microtome (Leica Microsystems GmbH, Wetzlar, Germany) and transferred to adhesive-coated slides. Immunohistochemistry was performed on these sections using a Leica BOND-III Fully Automated IHC and ISH Staining System (Leica Microsistemas S.L.U. All Microscopy and Histology, Barcelona, Spain) following the manufacturer's instructions. CD98hc expression was analyzed using the anti-CD98hc (Cell Signaling Technology, #47213) dilution 1:500, 20 minutes of incubation.

### Deletion of CD98hc by CRISPR/Cas9 and cell surface staining of CD98hc

MDA-MB231 cells (plate of 35 mm, 40-60 % confluence) were transfected with two plasmids (1.5 µg of CD98 CRISPR/Cas9 KO and 1.5 µg CD98 HDR) (Santa Cruz Biotechnology, sc-400501) using JetPEI DNA transfection reagent (Polyplustransfection SA, Illkirch, France) following the manufacturer’s instructions. Three days later, the cells were selected with 3 µg/ml of puromycin for two weeks (every 3 or 4 days the medium was renewed). The cells were maintained for two more weeks without puromycin and then single CD98 KO cells were selected and separated by flow cytometer. To do this, MDA-MB231 cells were treated with anti-CD98hc (10 nM) antibody during 20 minutes at 37°C. Then, the cells were trypsinized and collected in culture medium (DMEM+10% FBS). Next, the cells were centrifuged 1200 r.p.m. 5 minutes and resuspended in PBS + 2% BSA. Subsequently, cells were incubated with anti-Mouse FITC (1:100, Cytognos S.L., Salamanca, Spain) for 30 minutes in agitation at room temperature. Later, cells were washed twice PBS + 2% BSA and the expression of CD98hc was analyzed by cytometer (BD FACSAria ^TM^ III, BD transduction Laboratories). Cells without expression of CD98hc were separated individually in 96-well plates. The expression of CD98hc of the different clones was analyzed by western blot.

### Statistical analyses

Statistical analyses were performed using the software package SPSS 15.0 (SPSS Inc. Chicago, IL, USA). Comparison of continuous variables between two groups for in vitro assays were performed using a two-sided Student’s *t*-test. Differences were considered statistically significant when *p*<0.05. All experiments were repeated at least twice. Representative results of the findings are shown.

## Results

### Surface proteins differentially expressed in TNBC

**Fig. 1:**
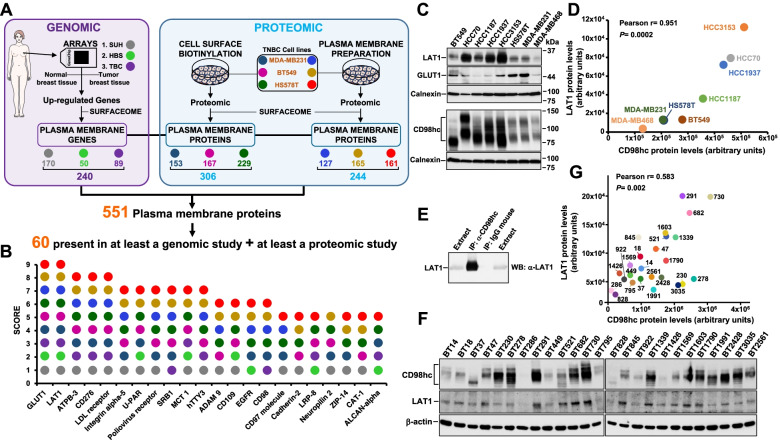
Identification of surface proteins differentially expressed in TNBC. **A** Schematic representation of the genomic and proteomic approaches. For the genomic approach, microarray data from normal and tumoral triple negative breast cancer of patients obtained by us (SUH) or deposited in databases (HBS and TBC) were used. The number of up-regulated cell surface proteins found using each dataset is shown. The proteomic approach was based on cell surface biotinylation and plasma membrane enrichment to detect surface proteins from MDA-MB231, BT549 and HS578T cells. Upon Orbitrap identification, plasma membrane proteins were selected using the Surfaceome database. **B** List of possible protein targets (score ≥5) ranked from highest to lowest score. The scoring criteria (one point per analysis) are described in the main text of this paper. The maximum score of 9, would be given to a protein identified in the three cell lines in the two proteomic methods and also identified in the three gene expression arrays. **C** Levels of expression of LAT1, CD98hc and GLUT1 in a panel of TNBC cell lines. Cell extracts of different TNBC cell lines were used to identified LAT1, CD98hc and GLUT1 by Western blot. Calnexin was used as a loading control. **D** Quantitation of expression of CD98hc and LAT1 of the experiment shown in (**C**). The graph represents the expression values of CD98hc and LAT1 for each cell line. Quantitation of CD98hc and LAT1 was made as described in the experimental procedures section. Pearson’s correlation coefficient and the *p* value are shown. **E** Co-immunoprecipitation studies of CD98hc and LAT1. One mg of HCC3153 extracts were immunoprecipitated with the anti-CD98hc antibody and the immunocomplexes were analyzed by Western with the anti-LAT1 antibody. Mouse IgG was used as a control. **F** Expression of CD98hc and LAT1 in tumoral samples of patients with TNBC. The tumours were homogenized and lysed. CD98hc and LAT1 were analyzed by Western blot. β-actin was used as a loading control. **G** Quantitation of expression of CD98hc and LAT1 of the experiment performed in (**F**). The graph represents the expression values of CD98hc and LAT1 for each tumor sample. Pearson’s correlation coefficient and the *p* value are shown

To identify surface proteins differentially expressed in TNBC, we used a combination of genomic and proteomic approaches (Fig. 1A and Supplementary Fig. 1). The genomic studies were performed using gene expression patient data freely available from two different studies (herewith termed TBC -Tokushima Breast Care Clinic study- [26], and HBS -Harvard Breast SPORE blood and tissue repository- [27]) or obtained from microarray analysis of patient samples from the Salamanca University Hospital (SUH). These datasets were selected because when we started our study, they were the only ones that included transcriptomic data from tumoral as well as normal breast tissue, allowing comparative analyses. Transcriptomic data were analyzed for genes differentially expressed in tumor versus normal breast tissue (Supplementary Fig. 1). Genes down regulated in the tumoral tissues versus normal tissues were eliminated, and those up-regulated analyzed for their plasma membrane location using the Surfaceome online database, which lists plasma membrane proteins (http://wlab.ethz.ch/surfaceome/) [31]. This filtering strategy yielded 170 (SUH), 50 (HBS) and 89 (TBC) transcripts, coding for a total of 240 membrane protein transcripts which were up-regulated in tumoral samples with respect to normal tissues (Supplementary Figs. 1, 2 and Supplementary Table 1). Of these, 60 transcripts were found to be present in at least two of the explored datasets, and 9 were present in the three datasets (Supplementary Fig. 2B).

The proteomic studies were based on two different experimental approaches: isolation of plasma membrane-enriched fractions and biotinylation of extracellularly exposed proteins. Plasma membrane enriched microsomal fractions were prepared from three prototypical TNBC cell lines (MDA-MB231, BT549 and HS578T) by ultracentrifugation (Fig. 1A and Supplementary Fig. 3A). The microsomal plasma membrane-enriched fractions were resuspended in a urea/thiourea solution, and the proteins identified by mass spectrometry. These studies led to the identification of 1865, 2074 and 2181 proteins in MDA-MB231, BT549 and HS578T cells, respectively (Supplementary Fig. 3B). Upon filtering using the Surfaceome database, these analyses led to the identification of 127, 165 and 161 plasma membrane proteins in MDA-MB231, BT549 and HS578T, respectively (Fig. 1A and Supplementary Fig. 3B). A total of 244 unique plasma membrane proteins were identified using this approach, with 77 of them present in all three cell lines (Supplementary Fig. 1 and Supplementary Fig. 3C). The second proteomic approach relied on cell surface biotinylation (Fig. 1A and Supplementary Fig. 4A) of proteins exposed to the extracellular milieu. Labelled proteins were precipitated with Streptavidin-Sepharose and ran 1 cm within 10% SDS-PAGE gels. A 1 cm^2^ gel slice was incubated in a protein digesting buffer containing trypsin, and proteolyzed protein fragments analyzed by mass spectrometry. These studies led to the identification of 396, 619 and 990 different proteins in MDA-MB231, BT549 and HS578T, respectively (Supplementary Fig. 4B), of which 153, 167 and 229 corresponded to plasma membrane proteins respectively (Supplementary Figs. 4B and C). A total of 306 proteins were identified, of which 70 were present in the three cell lines (Supplementary Fig. 1 and Supplementary Fig. 4C).

The combined analyses carried out using the genomic and proteomic studies identified a total of 551 plasma membrane proteins (Fig. 1A, Supplementary Table 1 and Supplementary Fig. 1). To explore potential ADC targets within them, proteins which were present in at least a genomic study and at least in a proteomic study were initially selected. Their identification in the genomic studies guaranteed differential expression in tumor tissues with respect to normal ones, while their detection in the proteomic studies carried out using cell lines should allow preclinical validation of their potentiality as ADC targets. Sixty proteins met those criteria (Fig. 1A and Supplementary Table 2). These proteins were then given a score depending on the number of analyses (nine in total: three genomic and six proteomic) in which they were present. Figure 1B shows the names of proteins that scored ≥5. Two of the proteins, the amino acid transporter LAT1 (*SLC7A5*) and the glucose transporter GLUT1 (*SLC2A1*) were found in the nine studies (Fig. 1B). Therefore, we chose them for initial assessment as potential as novel ADC targets. Western blotting analyses showed expression of LAT1 and GLUT1 in a panel of eight TNBC cell lines (Fig. 1C). Furthermore, functional studies showed that knocking down LAT1 or GLUT1 inhibited the proliferation of BT549 cells (Supplementary Figs. 5A and B). Knock down of GLUT1 also inhibited proliferation of MDA-MB231 cells (Supplementary Figs. 5C and D). However, knock down of LAT1 did not substantially affect the proliferation of MDA-MB231 cells.

As a next step in the evaluation of the potential use of LAT1 and GLUT1 as ADC targets, we searched for commercial sources of antibodies against those proteins, suitable to be prepared for use as ADCs. The antibodies should fulfill two properties. Firstly, they should be able to interact with the extracellular region of the human target and, secondly, be internalized. Exploration of commercial sources of antibodies using the Antibodypedia online tool led to the identification of an anti-SLC7A5/LAT1 antibody (BU53, Novus Biologicals) and an anti-GLUT1 antibody (MAB1418, R&D Systems, MN, USA) that could meet these properties. However, in our hands, those antibodies failed to either interact with the native extracellular region of these transporters and/or be internalized.

### The LAT1 partner CD98hc is overexpressed in TNBC

We reasoned that since LAT1 is part of a heterodimeric amino acid transport system, of which CD98hc represents the other partner, overexpression of LAT1 should be accompanied by increased levels of CD98hc. That could offer the interesting possibility of using CD98hc as an alternative ADC target, especially because most of that protein is exposed to the extracellular milieu. In line with the above hypothesis, CD98hc scored 6 in our classification system (Fig. 1B). Moreover, western blot analyses confirmed that CD98hc was expressed in all the TNBC cell lines studied (Fig. 1C). As expected, a linear correlation between the expression of LAT1 and CD98hc was observed (Fig. 1D), proving that expression of the two proteins is coordinated. Furthermore, immunoprecipitation studies carried out in the TNBC cell line HCC3153 confirmed the stable interaction between CD98hc and LAT1 (Fig. 1E).

**Fig. 2:**
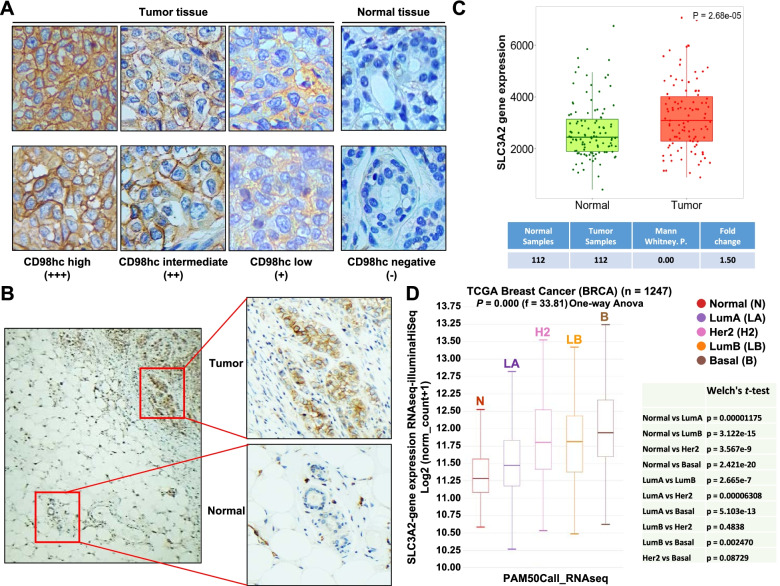
Expression of CD98hc in normal and TNBC tumoral tissue. **A** Immunohistochemical stainings of CD98hc showing tumor tissue scored as high (+++), intermediate (++) and low (+), and normal tissue with negative (-) staining. Magnification: 40X. **B** Immunohistochemical staining of CD98hc in a sample of a patient, showing staining of CD98hc only in tumor tissue and not in normal tissue. Magnification: 20 X. **C** Box plot showing *SLC3A2* gene expression levels in normal and tumor tissue of breast cancer patients. Data were obtained from the TNMplot database. **D** Box plot showing *SLC3A2* gene expression levels in different breast cancer subtypes from patients with breast cancer. The data were obtained using the Xena online tool

In patient samples, western blot studies showed CD98hc and LAT1 expression (Fig. 1F). In line with the data obtained using the TNBC cell lines, quantitative analyses also showed correlation between CD98hc and LAT1 expression (Fig. 1G). Immunohistochemical staining of TNBC tumor samples showed CD98hc expression in the tumoral tissue. As expected from the variable expression levels observed in the western analyses, different levels of staining of CD98hc (scored as high, intermediate, or low) were observed (Fig. 2A). Staining of CD98hc in normal breast tissue, when present in the same analyzed sample slice, was substantially lower than the staining present in adjacent tumoral cells (Fig. 2B). Comparative transcriptomic analyses of normal and tumoral breast tumors confirmed that CD98hc expression was significantly higher in tumoral tissues (Fig. 2C). Moreover, subtype specific analyses confirmed significantly higher levels of CD98hc in TNBC as compared to the normal-like phenotype (Fig. 2D).

### Efficient internalization of an antibody against CD98hc

**Fig. 3:**
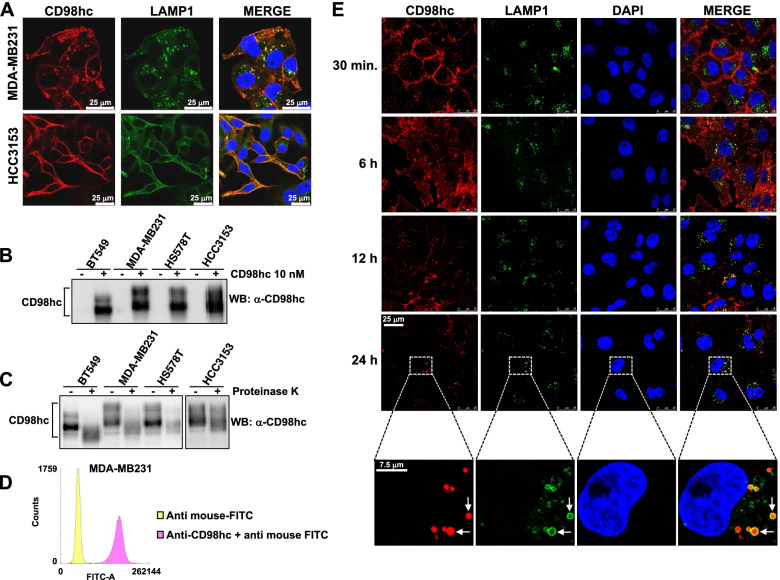
Internalization of an antibody against CD98hc. **A** Subcellular localization of CD98hc in MDA-MB231 and HCC3153 cells was analyzed by immunofluorescence. Scale bar = 25 μm. **B** Cell surface immunoprecipitation of CD98hc. The different TNBC cell lines treated or not with 10 nM of anti-CD98hc for 2 hours at 4°C were lysed and cell extracts precipitated with protein A-sepharose. CD98hc in those immunoprecipitates was analyzed by Western. **C** Protease protection experiments of CD98hc. Cells were treated with proteinase K, lysed and subjected to immunoprecipitation and Western analysis with the anti-CD98hc antibody. **D** FACS analyses of CD98hc cell surface expression. MDA-MB231 cells were incubated with 10 nM of anti-CD98hc for 20 minutes at 37°C. Cells were detached, incubated with an anti-mouse antibody conjugated to FITC, and fluorescence intensity was measured with a BD FACSAria ^TM^ III cytometer. The yellow histogram corresponds to signals from cells incubated with the secondary antibody alone, whereas the pink histogram represents the fluorescence due to the expression of CD98hc. **E** MDA-MB231 cells were seeded on coverslips and treated with 10 nM of anti-CD98hc for the times indicated. Scale bar = 25 μm. The images at the bottom of this section correspond to magnifications of a cell present in the images obtained at 24 hours. The white arrows indicate colocalization of CD98hc and LAMP1. Scale bar = 7.5 μm

Although the immunohistochemical staining pattern obtained in the TNBC samples was compatible with plasma membrane location of CD98hc, several additional studies were performed on cultured TNBC cell lines to explore the subcellular location of CD98hc. From six commercially available antibodies selected and tested, we chose the one that performed best in recognizing the native extracellular region of human CD98hc. Immunofluorescence experiments using that antibody revealed a pattern compatible with cell surface location of CD98hc (Fig. 3A). In fact, cell surface immunoprecipitation (Fig. 3B), protease protection experiments using proteinase K (Fig. 3C) as well as FACS staining (Fig. 3D) demonstrated that the epitope recognized by the anti-CD98hc antibody was exposed to the extracellular milieu. In addition to the characteristic surface staining, in some cell lines (e.g. MDA-MB231, Fig. 3A), a dotted pattern of staining was also present. Co-staining with several markers of intracellular organelles showed colocalization of CD98hc with the lysosomal marker LAMP1.

To explore the trafficking of the antibody recognizing the extracellular region of CD98hc, MDA-MB231, HCC3153 and BT549 cell lines were incubated in vivo for different times with a saturating dose (10 nM) of the anti-CD98hc ectodomain antibody. Immunofluorescence analyses showed that at 30 minutes of incubation, the anti-CD98hc antibody stained the cell surface (Fig. 3E and Supplementary Fig. 6). Extension of the incubation times for up to 24 hours showed a progressive and time-dependent shifting from the surface to a dotted staining pattern of the anti-CD98hc antibody. Colocalization studies showed that the intracellular dotted pattern was coincident with the lysosomal marker LAMP1 (Fig. 3E and Supplementary Figure 6).

### Construction and biological activity of an antibody-drug conjugate targeting CD98hc

**Fig. 4:**
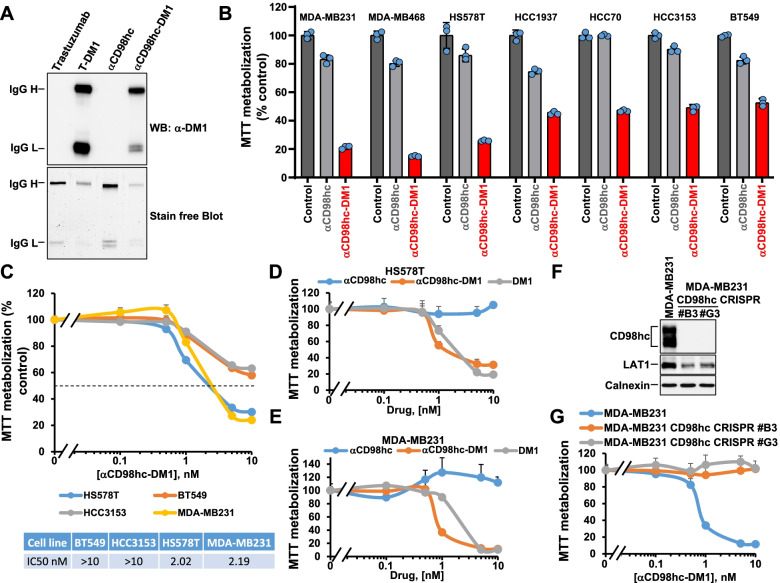
Generation and anti-proliferative activity of an antibody-drug conjugate targeting CD98hc. **A** Preparation of the antibody-drug conjugate targeting CD98hc. The coupling of DM1 to the anti-CD98hc antibody was analyzed by Western, by using an anti-DM1 antibody. Twenty nanograms of this ADC (αCD98hc-DM1), the nude anti-CD98hc (αCD98hc), trastuzumab or T-DM1 were loaded in 12% SDS-PAGE gels and analyzed for total protein (stain-free blot, lower image) and DM1 reactivity (upper panel). Trastuzumab and T-DM1 were used as a negative and positive controls. **B** Effect of anti-CD98hc-DM1 in a panel of TNBC cell lines. Cells were treated with anti-CD98hc and anti-CD98hc-DM1 10 nM for four days. The data are plotted as the percentage of MTT metabolization with respect to control. Results are shown as the mean ± SD of triplicates of an experiment repeated twice. **C** Dose-response analyses of the effect of anti-CD98hc-DM1 on four TNBC cell lines. Cells were treated with the ADC for four days at the indicated doses. The data are plotted as the percentage of MTT metabolization with respect to control. Results are shown as the mean ± SD of quadruplicates of an experiment repeated two times. **D** and **E** HS578T (**D**) and MDA-MB231 (**E**) cells were treated with anti-CD98hc, anti-CD98hc-DM1 or DM1 for four days at the indicated doses. The data are plotted as the percentage of MTT metabolization with respect to control. **F** Knockout of CD98hc in MDA-MB231 cells by CRISPR/Cas9. Parental MDA-MB231 cells and two different clones knocked out for CD98hc were lysed. The levels of expression of CD98hc and LAT1 were analyzed by Western blot. Calnexin was used as a loading control. **G** Dose-response analyses of the effect of anti-CD98hc-DM1 on parental and CD98hc CRISPR #B3, #G3 MDA-MB231 cells. Cells were treated with anti-CD98hc-DM1 for four days. Results are shown as the mean ± SD of quadruplicates of an experiment repeated three times

As the anti-CD98hc ectodomain antibody bound to native CD98hc on the cell surface and was capable of internalizing to the lysosomes, we used it as a skeleton to construct an anti-CD98hc ADC. Because of its proven effectiveness in the clinic [10], the antimicrotubular agent DM1 was used as a payload. To verify that DM1 was linked to the CD98hc antibody backbone, western blotting analyses of the uncoupled and coupled antibody were performed using an anti-DM1 antibody. As controls we used commercially available trastuzumab as well as trastuzumab-DM1 (T-DM1). These experiments demonstrated that DM1 was covalently coupled to both the heavy and light chains of the anti-CD98hc antibody, similarly to the commercially available T-DM1 (Fig. 4A).

To analyze the antiproliferative effect of anti-CD98hc-DM1, seven TNBC cell lines were treated with 10 nM of anti-CD98hc-DM1 or the anti-CD98hc nude antibody for 4 days. Treatment with the anti-CD98hc-DM1 antibody decreased MTT metabolization, used as a surrogate of cell number, of all the cell lines studied (Fig. 4B). Dose-response studies with anti-CD98hc-DM1 showed a dose-dependent decrease in MTT metabolization (Fig. 4C). Dose-response studies also showed that MDA-MB231 and HS578T cells responded similarly to anti-CD98hc-DM1 and the free payload DM1 (Figs. 4D and E). In contrast, the nude antibody did not present any substantial effect on the proliferation of MDA-MB231 and HS578T cells in concentrations up to 10 nM.

To analyze the specificity of the anti-CD98hc-DM1, CD98hc was eliminated in MDA-MB231 using the CRISPR/Cas9 technique (Fig. 4F). While in the parental MDA-MB231 cell line the ADC exerted a profound antiproliferative effect, the CD98hc-CRISPR clones (MDA-MB231 CD98hc CRISPR/Cas9 #B3 and #G3) were insensitive to the action of the anti-CD98hc-DM1 (Fig. 4G). Noteworthy, the clones without CD98hc grew much less than the parental line (Supplementary Fig. 7A), suggesting that CD98hc exerts a role in the control of cell proliferation. That conclusion was also supported by knock down experiments that showed that decreasing the levels of CD98hc inhibited proliferation (Supplementary Fig. 7B-E). Of note, no major effect on cell migration or adhesion was observed when the parental control cells were compared to the CRISPR/Cas9 #B3 or #G3 clones. LAT1 expression was found to be lower in CD98hc-CRISPR clones than in the parental MDA-MB231 cells (Fig. 4F).

### The anti-CD98hc-DM1 antibody provokes cell cycle arrest in mitosis and mitotic catastrophe

**Fig. 5:**
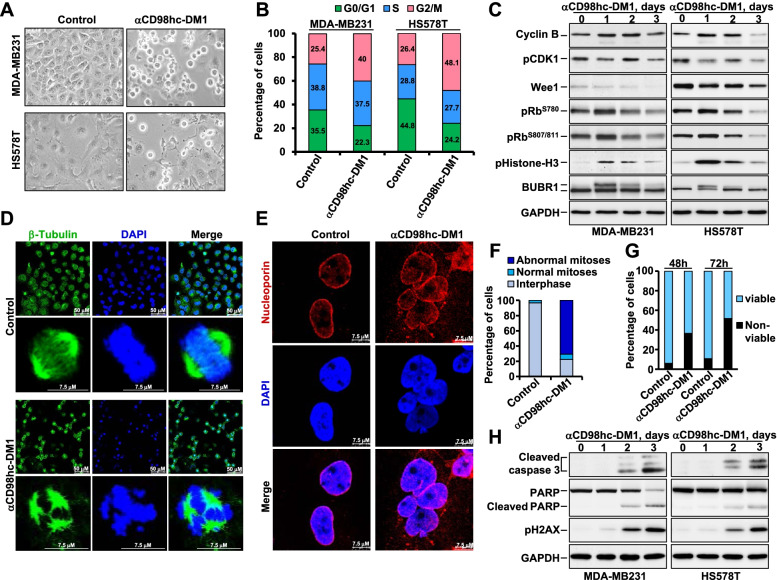
The anti-CD98hc-DM1 antibody provokes cell cycle arrest in mitosis and mitotic catastrophe. **A** Effect of anti-CD98-DM1 (10 nM, 24 hours) on the morphology of MDA-MB231 and HS578T cells grown as monolayers. The images were taken at 10X magnification. **B** Quantitative analyses of the action of anti-CD98hc-DM1 on the distribution of the different cell cycle phases in MDA-MB231 and HS578T cell lines. **C** MDA-MB231 and HS578T cells were treated with anti-CD98hc-DM1 (10 nM) and lysed at the indicated times. Analyses of the amounts of the different proteins studied were performed by Western blotting. GAPDH was used as loading control. **D** Effect of anti-CD98hc-DM1 on spindle assembly and organization. MDA-MB231 cells seeded on coverslips were treated with CD98hc-DM1 (10 nM) for 24 hours, fixed and stained. Scale bars are indicated. **E** Detection of giant multinucleated cells after anti-CD98hc-DM1 treatment. MDA-MB231 cells were treated with 10 nM anti-CD98hc-DM1 for 48 hours, fixed and stained for nucleoporin p62 (red) and DNA (blue). Scale bar = 7.5 μm. **F** Quantitation of abnormal mitoses, normal mitoses and interphase cells from the experiment shown in (**D**). **G** Bar graph representation of the percentage of viable (Annexin V-negative/PI-negative) and non-viable MDA-MB231 cells at 48 and 72 hours of treatment with 10 nM anti-CD98hc-DM1. **H** Effect of anti-CD98hc-DM1 on the levels of several apoptosis-related proteins. MDA-MB231 and HS578T cells were treated with 10 nM anti-CD98hc-DM1, lysed at 0, 1, 2, or 3 days and the indicated proteins analyzed by Western

Next, the mechanism responsible for the antiproliferative activity of the anti-CD98hc-DM1 antibody was studied. The ADC provoked rounding of MDA-MB231 or HS578T cells after 24 hours of treatment (Fig. 5A), which may be indicative of accumulation of cells in mitosis and/or induction of apoptosis. Propidium iodide staining showed that treatment with the ADC caused accumulation of the cells in the G2/M phase of the cell cycle (Fig. 5B and Supplementary Figure 8). A concomitant decrease of cells in G0/G1 was observed. To analyze whether anti-CD98hc-DM1 arrested cells in G2 or M, MDA-MB231 and HS578T cells were treated with anti-CD98hc-DM1 for 0, 1, 2 and 3 days and proteins involved in different cell cycle phases analyzed. Treatment with the ADC caused accumulation of the mitotic markers pHistone-H3 and pBUBR1, reaching a peak at one day of treatment (Fig. 5C). Moreover, at that time of treatment with anti-CD98hc-DM1, dephosphorylation of CDK1 and a decrease of Wee1, both events required for entry of cells in mitosis [32], were detected. An increase in Cyclin B, which acts partnering with CDK1 to allow entry of cells in mitosis and progression along prophase and metaphase [33–35], was also observed. An increase in Rb phosphorylation, consistent with a state of hyperphosphorylation of Rb until late mitosis, was also detected in cells treated for one day with the ADC [36, 37].

Immunofluorescence analyses showed that anti-CD98hc-DM1 provoked appearance of aberrant mitotic spindles at 24 hours of treatment (Figs. 5D and F). Immunofluorescence analyses also demonstrated the appearance of giant multinucleated cells at 48 hours of treatment with anti-CD98hc-DM1 (Fig. 5E). The presence of these multinucleated cells, likely caused by deficient spindle assembly due to the maytansinoid derivative, was indicative of mitotic catastrophe, a form of cell death triggered by deficient mitotic progression [38, 39]. Annexin V/propidium iodide staining experiments indicated that anti-CD98hc-DM1 was able to provoke a substantial increase in apoptotic cell death at 48 and 72 hours of incubation with anti-CD98hc-DM1 (Fig 5G). Moreover, treatment of MDA-MB231 and HS578T cells with anti-CD98hc-DM1 caused an increase in cleaved forms of caspase 3 and PARP (Fig. 5H) together with an increase in the levels of the DNA damage marker pH2AX, at times that paralleled the changes in cleaved caspase 3 and PARP. Together, these data indicate that treatment of TNBC cell lines with anti-CD98hc-DM1 cause cell cycle arrest which progresses into cell death.

### The CD98hc-directed ADC shows in vivo antitumoral activity and potentiates the action of standard of care drugs

**Fig. 6:**
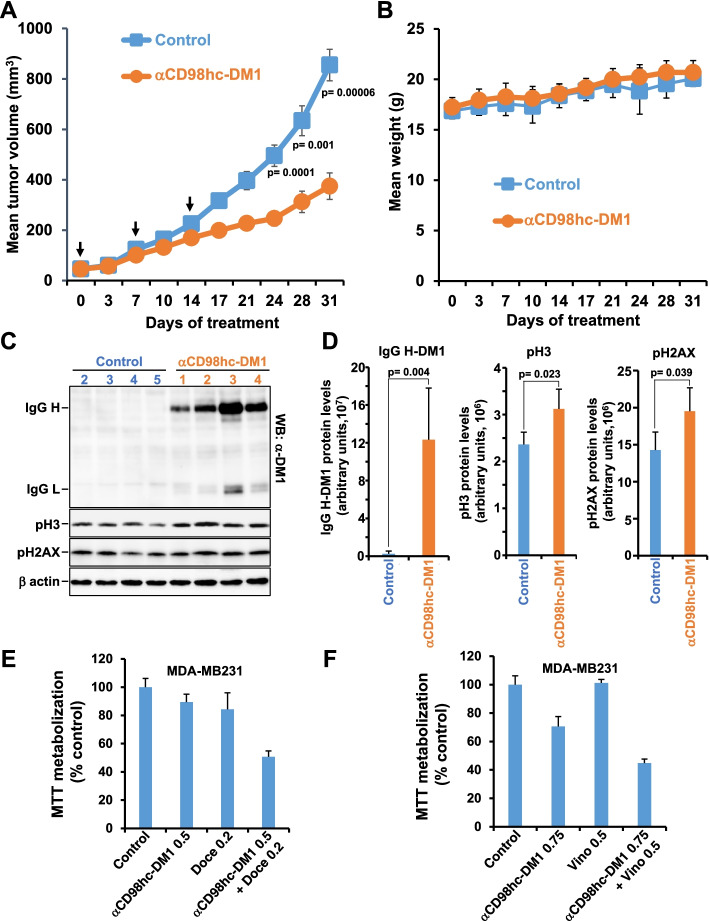
The anti-CD98hc-DM1 has antitumoral activity in vivo and potentiates the action of standard of care drugs. **A** Analysis of the “in vivo” effect of anti-CD98hc-DM1 on tumor growth in nude mice implanted with MDA-MB231 cells. Arrows indicate days of administration of anti-CD98hc-DM1. Data are plotted as mean tumor volumes ± SEM. *P* values were calculated using Student *t* test (two-sided). **B** Effect of CD98hc-DM1 on the weight of mice. Data are plotted as mean ± SD of six mice/group. **C** Analyses of DM1, pH3 and pH2AX levels in tumors from mice. Seventeen days after the last treatment, tumors were resected and immediately frozen in liquid nitrogen. Expression of DM1, pH3 and pH2AX was analyzed by Western. β-actin was used as a loading control. **D** Quantitation of the levels of DM1, pH3 and pH2AX of the experiment performed in (**C**). The graphs represent the mean ± S.D of the different proteins (arbitrary units) of control and treated (anti-CD98hc-DM1) groups. Student’s *t* test was used to analyze differences among groups. (**E** and **F**) MDA-MB231 cells were treated with CD98hc-DM1 alone or in combination with Docetaxel (**E**) or Vinorelbine (**F**) for four days at the indicated doses (nM)

The in vivo antitumoral effect of anti-CD98hc-DM1 was investigated. Mice were injected with MDA-MB231 cells in the caudal mammary fat pad and when tumors reached a mean volume of 45 mm^3^, mice were randomized to receive vehicle or anti-CD98hc-DM1 intraperitoneally every week (three doses in total). Treatment with anti-CD98hc-DM1 produced a significant reduction in tumor growth (Fig. 6A). Body weights of the treated and control mice were similar throughout the experiment (Fig. 6B). Tumors from control or anti-CD98hc-DM1-treated mice were removed at the end of the experiment (two weeks after the last treatment). The anti-CD98hc-DM1 was detected only in tumors from treated mice (Figs. 6C and D). In addition, increases in pH3 and pH2AX were detected in mice treated with the ADC.

Most antitumoral treatments are based on drug combinations which have clearly demonstrated superior antitumoral activities as compared to single treatments [6]. Because of this, we decided to explore whether the anti-CD98hc-DM1 potentiated the action of drugs used in the therapy of TNBC. Combinations of docetaxel or vinorelbine with anti-CD98hc-DM1 were more efficient than treatment with the individual drugs (Figs. 6E and F).

## Discussion

The present study was designed to elaborate a catalog of membrane proteins present in TNBC with the final objective of using that information for therapeutic purposes. To that end, genomic as well as proteomic strategies were used. The genomic studies were designed to select those proteins that were differentially expressed in the tumoral samples with respect to normal breast tissue. The proteomic studies exclusively relied on the analyses of surface proteins present in cell lines. These studies led to the identification of a substantial number of cell surface proteins, which still likely underestimates the real number of membrane proteins expressed in TNBC. To select those proteins more frequently overexpressed, a ranking method was established. A prerequisite was that a candidate protein would be found in one of the genomic studies. That was important as the genomic studies were focused at identifying proteins upregulated in tumoral tissues when compared to normal breast tissue. The second condition that was imposed required the expression of the protein in at least one of the proteomic studies. That characteristic was important with the purpose of preclinically validating the potential therapeutic value of the protein target. Such filtering strategy resulted in the generation of a restricted list of 60 different proteins. Of them, the membrane transporters LAT1 and GLUT1 appeared overexpressed in the nine studies performed. We succeeded in finding antibodies that allowed the analyses of expression of these proteins by western. However, attempts to find antibodies that could be used as backbones to prepare ADCs against those proteins failed. One of the potential reasons for that may rely in the structure of these proteins. In fact, they present short amino acid loops in the parts of the protein facing the extracellular media, and that may impose difficulties in creating good antibodies recognizing the extracellular region of LAT1 or GLUT1.

A totally different situation was presented in the case of the LAT1 heterodimeric partner CD98hc. The latter is an 80 kDa type II transmembrane protein that exposes most of its sequence to the extracellular region [14, 15]. CD98hc is heavily glycosylated, and that circumstance appears to be responsible for its heterogeneous molecular weight. The fact that LAT1 was overexpressed in TNBC and is stably bound to CD98hc raised the possibility that expression of the latter in TNBC could also be higher than its expression in normal breast tissue. In fact, Furuya et al have reported that CD98hc and LAT1 are co-expressed at a high rate in TNBC and the expression of these proteins was not observed in normal mammary ducts [40]. On the other hand, CD98hc was discovered by mRNA screening to identify membrane proteins overexpressed in invasive breast cancer [41]. Our genomic and proteomic studies fall in line with those already published, confirming overexpression of CD98hc in TNBC.

Antibodies that interacted with the extracellular region of human CD98hc were found and they allowed the study of the internalization properties of membrane-exposed CD98hc in several TNBC cell lines. Immunofluorescence staining of these cell lines demonstrated the expression of the protein at the cell surface. In addition, a dotted pattern of staining was also observed. The coincidence of that dotted pattern with the lysosomal marker LAMP1 suggested that CD98hc could be directed to the lysosomes, and that made it a candidate for the development of an ADC. Formerly, it has been described that the lysosomal protein LAPTM4b recruits LAT1-CD98hc to lysosomes [42]. Moreover, dynamic trafficking experiments using an anti-CD98hc antibody that reacted with its extracellular domain demonstrated that the antibody-CD98hc could be targeted to the lysosomes, the site where ADCs are processed intracellularly [43, 44].

Construction of an ADC targeting CD98hc was based on the anti-CD98hc used for the immunofluorescence studies, to which the antitubular agent DM1 was coupled. The resulting anti-CD98hc-DM1 exerted a strong antiproliferative effect in all seven TNBC cell lines tested. In contrast, the nude antibody did not have much effect on the proliferation of the in vitro TNBC models. That the action of the anti-CD98hc-ADC was specifically mediated by its interaction with CD98hc was demonstrated by using loss of function models created by elimination of CD98hc by CRISPR/Cas9. Especially relevant was the in vivo evidence of the antitumoral action of the CD98hc-ADC. In mice orthotopically injected with TNBC cells, treatment with the anti-CD98hc-ADC provoked a substantial and statistically significant inhibition of tumor growth with a limited number of treatments. Western blotting studies proved accumulation of the anti-CD98hc-ADC in tumoral tissues. That is important from the point of view of selective action of the anti-CD98hc-ADC on tumoral cells. Moreover, no overt signs of toxicity were found, including analyses of body weight in animals treated with the ADC. However, it should also be mentioned that one of the limitations of the present study is the fact that the anti-CD98hc-ADC recognizes the human version of CD98hc and does not interact with avidity with the murine version. That situation impedes fair assessment of the potential toxicities of the anti-CD98hc-ADC due to specific action on endogenous CD98hc.

The studies reported here accomplished several objectives. On one side, we describe a strategy that allowed the identification of a number of membrane proteins overexpressed in TNBC (graphically summarized in Supplementary Fig. 9). The catalog of membrane proteins provided may allow further analyses of their potentiality as therapeutic targets. Moreover, the identification of CD98hc as an overexpressed protein in TNBC, together with its internalization and lysosomal targeting properties, and the antitumoral capability of an ADC against this protein, demonstrate the robustness of the approach herewith reported to identify novel potential targets for therapeutic intervention in TNBC. The encouraging preclinical data with the anti-CD98hc-ADC open the door to the exploration of its potential use in the clinic. Moreover, the possibility that CD98hc could be overexpressed in other tumoral types should be explored. If that were the case, that circumstance would extend further the value of the findings herewith reported, by opening the possibility of using CD98hc as a novel ADC therapeutic target for the therapy of other tumoral diseases. Finally, the description of this approach together with its validation in the case of one of the antigens identified, establish the bases for the application of the strategy herewith followed to define novel potential ADC targets in other solid or hematological tumors. We are in fact using it to uncover novel targets of ADCs in colorectal cancer.

## Conclusions

By using a multiomic approach, we were able to generate a list of potential ADC targets in TNBC. In addition to offering a list of membrane proteins that may be suitable for targeting with ADCs, the validation of CD98hc as an ADC target sustains the robustness of the strategy used to identify membrane proteins that can represent ADC targets. From a more specific point of view, the data obtained with the anti-CD98hc-ADC raise the possibility of targeting that membrane protein for the treatment of patients with TNBC.

## Supplementary Information

**Additional file 1: Supplementary Figure 1.** Schematic flow chart representation of the genomic and proteomic approaches used to identify cell surface proteins in TNBC. **Supplementary Figure 2.** A) The table shows the data generated form the microarray analyses to identify cell surface proteins upregulated in TNBC. B) Venn diagram showing the number of genes specifically identified in each array and those that are common among them. **Supplementary Figure 3.** A) Procedure used to obtain enriched plasma membrane microsomal fraction, used to identify plasma membrane proteins in TNBC cell lines. B) The table shows the total number of proteins identified, as well as those that correspond to plasma membrane proteins. C) Venn diagram showing the number of proteins specifically identified in each cell line and those that are common among them. **Supplementary Figure 4.** A) Schematic representation of the protocol used in cell surface biotinylation experiments. B) The table shows the proteins identified and those that correspond to plasma membrane proteins. C) Venn diagram showing the number of proteins identified in each cell line and those that are common among them. **Supplementary Figure 5.** BT549 (A and B) and MDA-MB231 (C and D) cells were infected with lentivirus containing the shRNA control (sh-Control) or the shRNA sequences targeting GLUT1 or LAT1. Knockdown efficiency was verified by western (A and C), and the effect of the knockdowns on cell proliferation was analyzed by MTT metabolization (B and D). GAPDH was used as a loading control. **Supplementary Figure 6.** BT549 and HCC3153 cells were seeded on coverslips and treated with 10 nM of anti-CD98hc for the indicated times. Cells were fixed and stained for CD98hc (red), LAMP1 (green) and DNA (blue). Scale bar = 25 μm. Magnification of one cell at 24 hours of treatment is shown. Scale bar = 10 and 7.5 μm. **Supplementary Figure 7.** A) Dose-response analyses of the anti-proliferative effect of anti-CD98hc-DM1 in MDA-MB231 CD98hc CRISPR #B3, #G3 and parental MDA-MB231 cells. Cells were treated with anti-CD98hc-DM1 for four days at the indicated doses. Results are shown as the mean ± SD of quadruplicates of an experiment repeated three times. B and D) BT549 (B) and MDA-MB231 (D) cells were infected with lentivirus containing the shRNA control (sh-Control) or two shRNA sequences targeting CD98hc (sh-CD98hc #3 and #7). To verify the knockdown efficiency, levels of CD98hc were analyzed by Western blot. Calnexin was used as a loading control. C and E) BT549 (C) and MDA-MB231 (E) cells infected with lentivirus containing the shRNA control (sh-Control) or two shRNA sequences targeting CD98hc were plated and the MTT metabolization was measured at the times indicated. **Supplementary Figure 8.** Cell cycle profiles of TNBC cells treated with CD98hc-DM1. Cells were treated for one day with CD98hc-DM1 (10 nM), and then harvested and stained with propidium iodide for cell cycle analysis, following the procedure described in the materials and methods section. **Supplementary Figure 9.** Graphical representation of the surfaceome and the strategy to develop ADCs against differentially expressed proteins. Genomic as well as proteomic strategies allow the identification of proteins overexpressed or newly expressed by tumors with respect to normal tissue. That information may be used to develop an antibody that targets the differentially expressed protein, and that may be used as a backbone for the preparation of an ADC. Once prepared, in vitro and in vivo models can be used to define the antitumoral activity of the ADC as well as its mechanism of action.

**Additional file 2. **

**Additional file 3. **

## Data Availability

The SUH array obtained at our University Hospital in Salamanca is available in the NCBI GEO database with accession no. GSE185645.

## Abbreviations

TNBC: Triple negative breast cancer
ADCs: Antibody-drug conjugates
DMEM: Dulbecco’s modified Eagle medium
FBS: Fetal bovine serum
TBC: Tokushima Breast care Clinic study
HBS: Harvard Breast SPORE blood and tissue repository
SUH: Salamanca University Hospital
NCBI: National Center for Biotechnology Information
T-DM1: Trastuzumab-emtansine

## References

[1] DeSantis C, Ma J, Bryan L, Jemal A (2014). Breast cancer statistics, 2013. CA Cancer J Clin.

[2] Sisti A, Huayllani MT, Boczar D, Restrepo DJ, Spaulding AC, Emmanuel G (2020). Breast cancer in women: a descriptive analysis of the national cancer database. Acta Biomed.

[3] Won KA, Spruck C (2020). Triplenegative breast cancer therapy: current and future perspectives (Review). Int J Oncol.

[4] Gupta GK, Collier AL, Lee D, Hoefer RA, Zheleva V, Siewertsz van Reesema LL (2020). Perspectives on triple-negative breast cancer: current treatment strategies, unmet needs, and potential targets for future therapies. Cancers (Basel)..

[5] Avalos-Moreno M, Lopez-Tejada A, Blaya-Canovas JL, Cara-Lupianez FE, Gonzalez-Gonzalez A, Lorente JA (2020). Drug repurposing for triple-negative breast cancer. J Pers Med..

[6] Ocana A, Pandiella A (2017). Targeting oncogenic vulnerabilities in triple negative breast cancer: biological bases and ongoing clinical studies. Oncotarget.

[7] Waks AG, Winer EP (2019). Breast cancer treatment: a review. JAMA.

[8] Schmid P, Rugo HS, Adams S, Schneeweiss A, Barrios CH, Iwata H (2020). Atezolizumab plus nab-paclitaxel as first-line treatment for unresectable, locally advanced or metastatic triple-negative breast cancer (IMpassion130): updated efficacy results from a randomised, double-blind, placebo-controlled, phase 3 trial. Lancet Oncol.

[9] Schmid P, Adams S, Rugo HS, Schneeweiss A, Barrios CH, Iwata H (2018). Atezolizumab and Nab-Paclitaxel in advanced triple-negative breast cancer. N Engl J Med.

[10] Garcia-Alonso S, Ocana A, Pandiella A (2020). Trastuzumab emtansine: mechanisms of action and resistance, clinical progress, and beyond. Trends Cancer..

[11] Bardia A, Mayer IA, Vahdat LT, Tolaney SM, Isakoff SJ, Diamond JR (2019). Sacituzumab Govitecan-hziy in refractory metastatic triple-negative breast cancer. N Engl J Med.

[12] Bardia A, Mayer IA, Diamond JR, Moroose RL, Isakoff SJ, Starodub AN (2017). Efficacy and safety of anti-trop-2 antibody drug conjugate Sacituzumab Govitecan (IMMU-132) in heavily pretreated patients with metastatic triple-negative breast cancer. J Clin Oncol.

[13] Rugo HS, Bardia A, Tolaney SM, Arteaga C, Cortes J, Sohn J (2020). TROPiCS-02: a phase III study investigating sacituzumab govitecan in the treatment of HR+/HER2- metastatic breast cancer. Future Oncol.

[14] Cantor JM, Ginsberg MH (2012). CD98 at the crossroads of adaptive immunity and cancer. J Cell Sci.

[15] Deves R, Boyd CA (2000). Surface antigen CD98(4F2): not a single membrane protein, but a family of proteins with multiple functions. J Membr Biol.

[16] Nakamura E, Sato M, Yang H, Miyagawa F, Harasaki M, Tomita K (1999). 4F2 (CD98) heavy chain is associated covalently with an amino acid transporter and controls intracellular trafficking and membrane topology of 4F2 heterodimer. J Biol Chem.

[17] Ortiz-Ruiz MJ, Alvarez-Fernandez S, Parrott T, Zaknoen S, Burrows FJ, Ocana A (2014). Therapeutic potential of ERK5 targeting in triple negative breast cancer. Oncotarget.

[18] Montero JC, Pandiella A (2021). PDCD4 limits prooncogenic neuregulin-ErbB signaling. Cell Mol Life Sci.

[19] Esparis-Ogando A, Alegre A, Aguado B, Mateo G, Gutierrez N, Blade J (2005). Bortezomib is an efficient agent in plasma cell leukemias. Int J Cancer.

[20] Montero JC, Yuste L, Diaz-Rodriguez E, Esparis-Ogando A, Pandiella A (2002). Mitogen-activated protein kinase-dependent and -independent routes control shedding of transmembrane growth factors through multiple secretases. Biochem J.

[21] Esparis-Ogando A, Diaz-Rodriguez E, Montero JC, Yuste L, Crespo P, Pandiella A (2002). Erk5 participates in neuregulin signal transduction and is constitutively active in breast cancer cells overexpressing ErbB2. Mol Cell Biol.

[22] Cabrera N, Diaz-Rodriguez E, Becker E, Martin-Zanca D, Pandiella A (1996). TrkA receptor ectodomain cleavage generates a tyrosine-phosphorylated cell-associated fragment. J Cell Biol.

[23] Massague J (1983). Epidermal growth factor-like transforming growth factor .I. Isolation, chemical characterization, and potentiation by other transforming factors from feline sarcoma virus-transformed rat cells. J Biol Chem..

[24] Diaz-Rodriguez E, Cabrera N, Esparis-Ogando A, Montero JC, Pandiella A (1999). Cleavage of the TrkA neurotrophin receptor by multiple metalloproteases generates signalling-competent truncated forms. Eur J Neurosci.

[25] Seoane S, Montero JC, Ocana A, Pandiella A (2016). Breast cancer dissemination promoted by a neuregulin-collagenase 3 signalling node. Oncogene.

[26] Komatsu M, Yoshimaru T, Matsuo T, Kiyotani K, Miyoshi Y, Tanahashi T (2013). Molecular features of triple negative breast cancer cells by genome-wide gene expression profiling analysis. Int J Oncol.

[27] Richardson AL, Wang ZC, De Nicolo A, Lu X, Brown M, Miron A (2006). X chromosomal abnormalities in basal-like human breast cancer. Cancer Cell.

[28] Li C, Wong WH (2001). Model-based analysis of oligonucleotide arrays: expression index computation and outlier detection. Proc Natl Acad Sci U S A.

[29] Irizarry RA, Hobbs B, Collin F, Beazer-Barclay YD, Antonellis KJ, Scherf U (2003). Exploration, normalization, and summaries of high density oligonucleotide array probe level data. Biostatistics.

[30] Ritchie ME, Phipson B, Wu D, Hu Y, Law CW, Shi W (2015). limma powers differential expression analyses for RNA-sequencing and microarray studies. Nucleic Acids Res..

[31] Bausch-Fluck D, Goldmann U, Muller S, van Oostrum M, Muller M, Schubert OT (2018). The in silico human surfaceome. Proc Natl Acad Sci U S A.

[32] Lapenna S, Giordano A (2009). Cell cycle kinases as therapeutic targets for cancer. Nat Rev Drug Discov.

[33] Solomon MJ, Glotzer M, Lee TH, Philippe M, Kirschner MW (1990). Cyclin activation of p34cdc2. Cell.

[34] Pines J (2006). Mitosis: a matter of getting rid of the right protein at the right time. Trends Cell Biol.

[35] Lindqvist A, van Zon W, Karlsson Rosenthal C, Wolthuis RM (2007). Cyclin B1-Cdk1 activation continues after centrosome separation to control mitotic progression. PLoS Biol..

[36] Weinberg RA (1995). The retinoblastoma protein and cell cycle control. Cell.

[37] Claudio PP, De Luca A, Howard CM, Baldi A, Firpo EJ, Koff A (1996). Functional analysis of pRb2/p130 interaction with cyclins. Cancer Res.

[38] Barok M, Joensuu H, Isola J (2014). Trastuzumab emtansine: mechanisms of action and drug resistance. Breast Cancer Res.

[39] Barok M, Tanner M, Koninki K, Isola J (2011). Trastuzumab-DM1 causes tumour growth inhibition by mitotic catastrophe in trastuzumab-resistant breast cancer cells in vivo. Breast Cancer Res.

[40] Furuya M, Horiguchi J, Nakajima H, Kanai Y, Oyama T (2012). Correlation of L-type amino acid transporter 1 and CD98 expression with triple negative breast cancer prognosis. Cancer Sci.

[41] Esseghir S, Reis-Filho JS, Kennedy A, James M, O'Hare MJ, Jeffery R (2006). Identification of transmembrane proteins as potential prognostic markers and therapeutic targets in breast cancer by a screen for signal sequence encoding transcripts. J Pathol.

[42] Milkereit R, Persaud A, Vanoaica L, Guetg A, Verrey F, Rotin D (2015). LAPTM4b recruits the LAT1-4F2hc Leu transporter to lysosomes and promotes mTORC1 activation. Nat Commun.

[43] Erickson HK, Park PU, Widdison WC, Kovtun YV, Garrett LM, Hoffman K (2006). Antibody-maytansinoid conjugates are activated in targeted cancer cells by lysosomal degradation and linker-dependent intracellular processing. Cancer Res.

[44] Tsuchikama K, An Z (2018). Antibody-drug conjugates: recent advances in conjugation and linker chemistries. Protein Cell.

