# Supplementary material for: Surfaceome analyses uncover CD98hc as an antibody drug-conjugate target in triple negative breast cancer
Source: J Exp Clin Cancer Res. 2022 Mar 22;41:106. doi: 10.1186/s13046-022-02330-4 (PMC8941813; doi:10.1186/s13046-022-02330-4)
Supplement: Supplementary file 1 — Additional file 1: Supplementary Figure 1. Schematic flow chart representation of the genomic and proteomic approaches used to identify cell surface proteins in TNBC. Supplementary Figure 2. A) The table shows the data generated form the microarray analyses to identify cell surface proteins upregulated in TNBC. B) Venn diagram showing the number of genes specifically identified in each array and those that are common among them. Supplementary Figure 3. A) Procedure used to obtain enriched plasma membrane microsomal fraction, used to identify plasma membrane proteins in TNBC cell lines. B) The table shows the total number of proteins identified, as well as those that correspond to plasma membrane proteins. C) Venn diagram showing the number of proteins specifically identified in each cell line and those that are common among them. Supplementary Figure 4. A) Schematic representation of the protocol used in cell surface biotinylation experiments. B) The table shows the proteins identified and those that correspond to plasma membrane proteins. C) Venn diagram showing the number of proteins identified in each cell line and those that are common among them. Supplementary Figure 5. BT549 (A and B) and MDA-MB231 (C and D) cells were infected with lentivirus containing the shRNA control (sh-Control) or the shRNA sequences targeting GLUT1 or LAT1. Knockdown efficiency was verified by western (A and C), and the effect of the knockdowns on cell proliferation was analyzed by MTT metabolization (B and D). GAPDH was used as a loading control. Supplementary Figure 6. BT549 and HCC3153 cells were seeded on coverslips and treated with 10 nM of anti-CD98hc for the indicated times. Cells were fixed and stained for CD98hc (red), LAMP1 (green) and DNA (blue). Scale bar = 25 μm. Magnification of one cell at 24 hours of treatment is shown. Scale bar = 10 and 7.5 μm. Supplementary Figure 7. A) Dose-response analyses of the anti-proliferative effect of anti-CD98hc-DM1 in MDA-MB231 CD98hc CRI [file 13046_2022_2330_MOESM1_ESM.pdf]

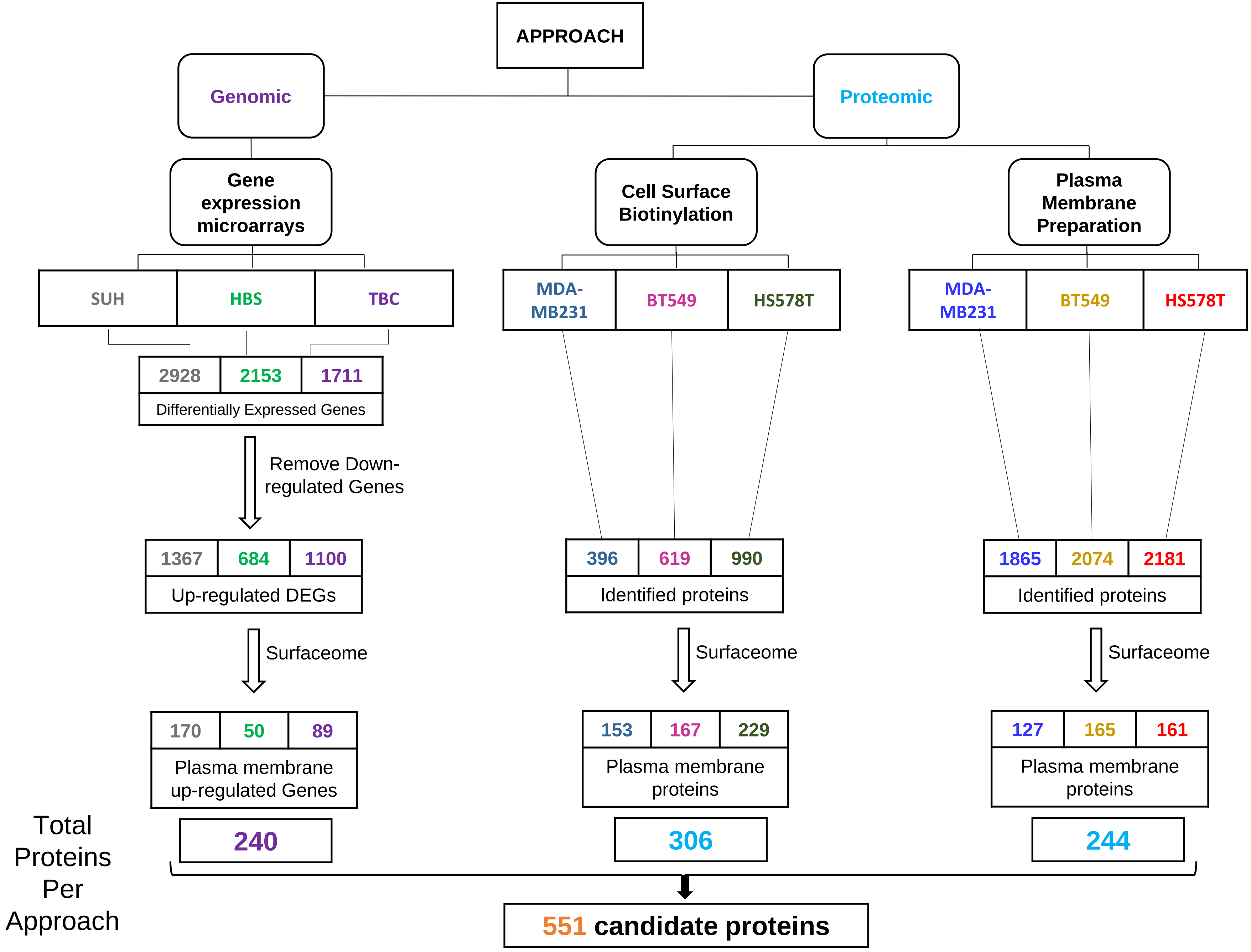

Supplementary Figure 1

A

Array	Differentially expressed genes	Up-regulated genes	Plasma membrane up-regulated genes
SUH	2928	1367	170
HBS	2153	684	50
TBC	1711	1100	89

B

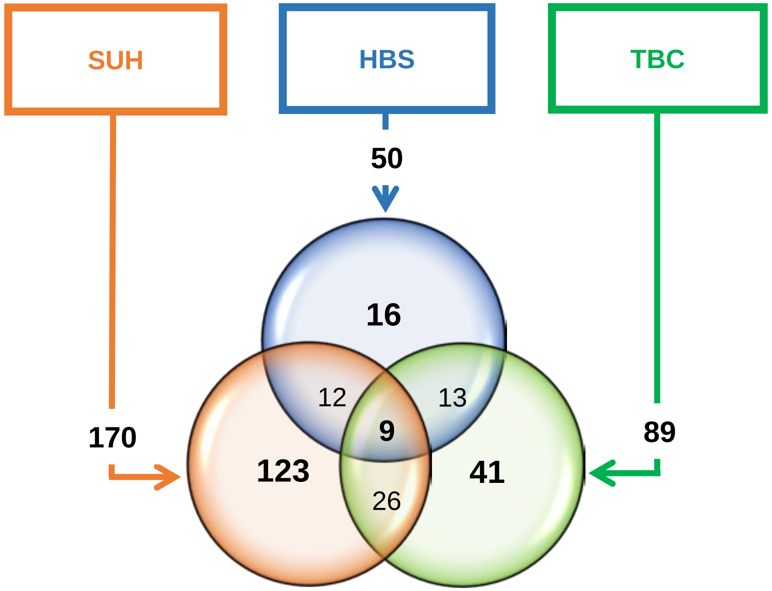

Supplementary Figure 2

A

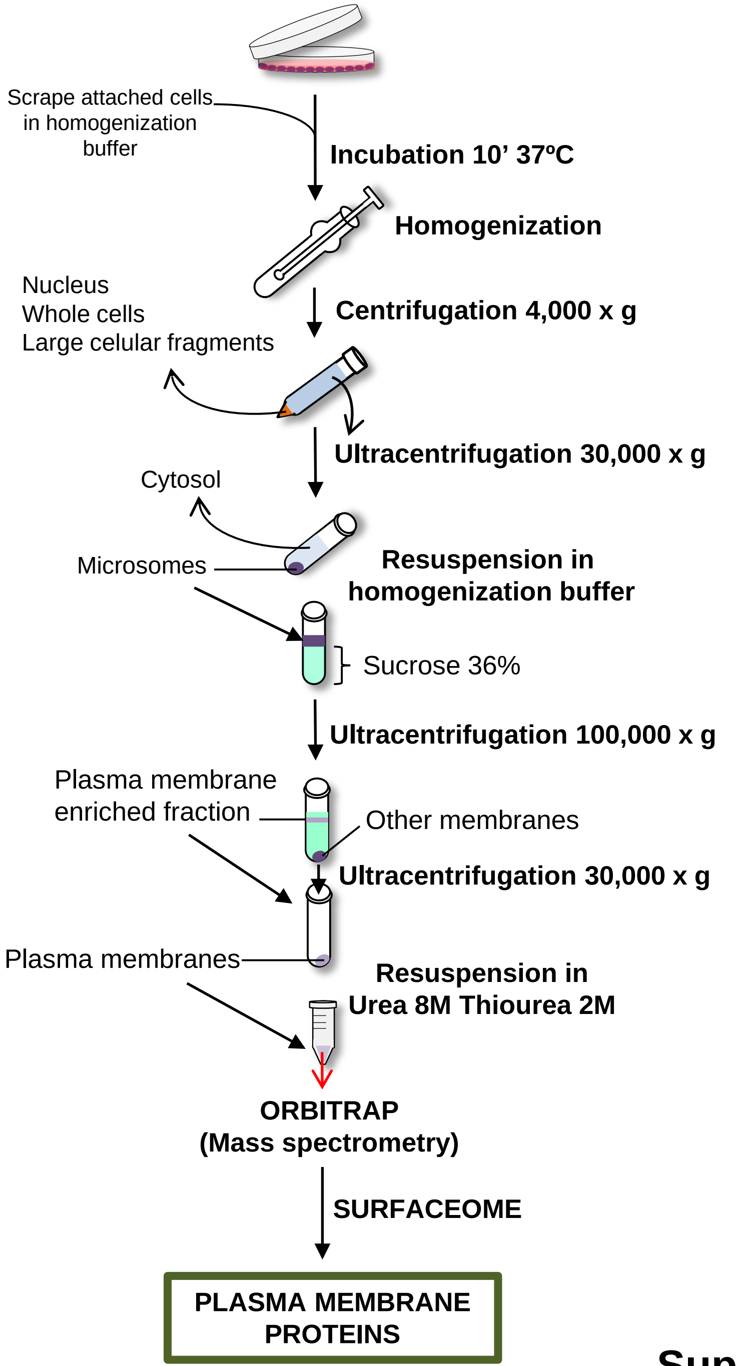

B

Cell line	Identified proteins	PLASMA MEMBRANE PROTEINS
MDA-MB-231	1865	127
BT549	2074	165
HS578T	2181	161

C

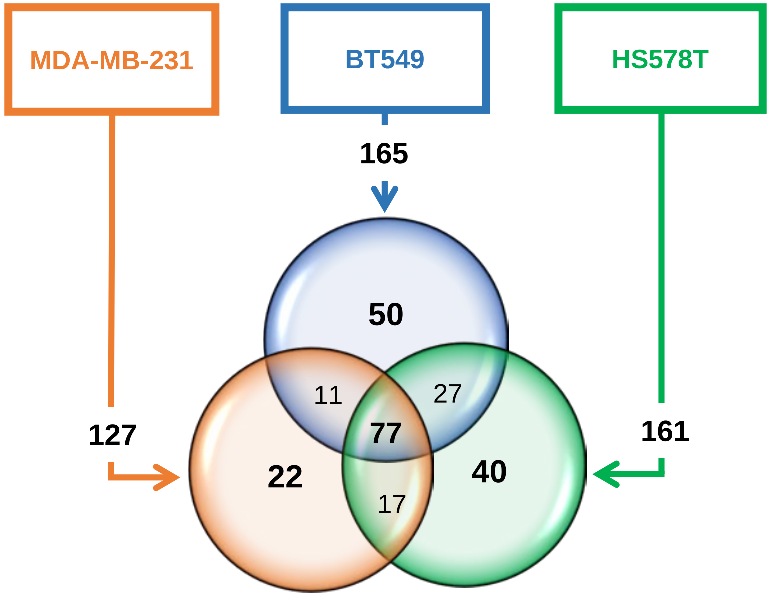

Supplementary Figure 3

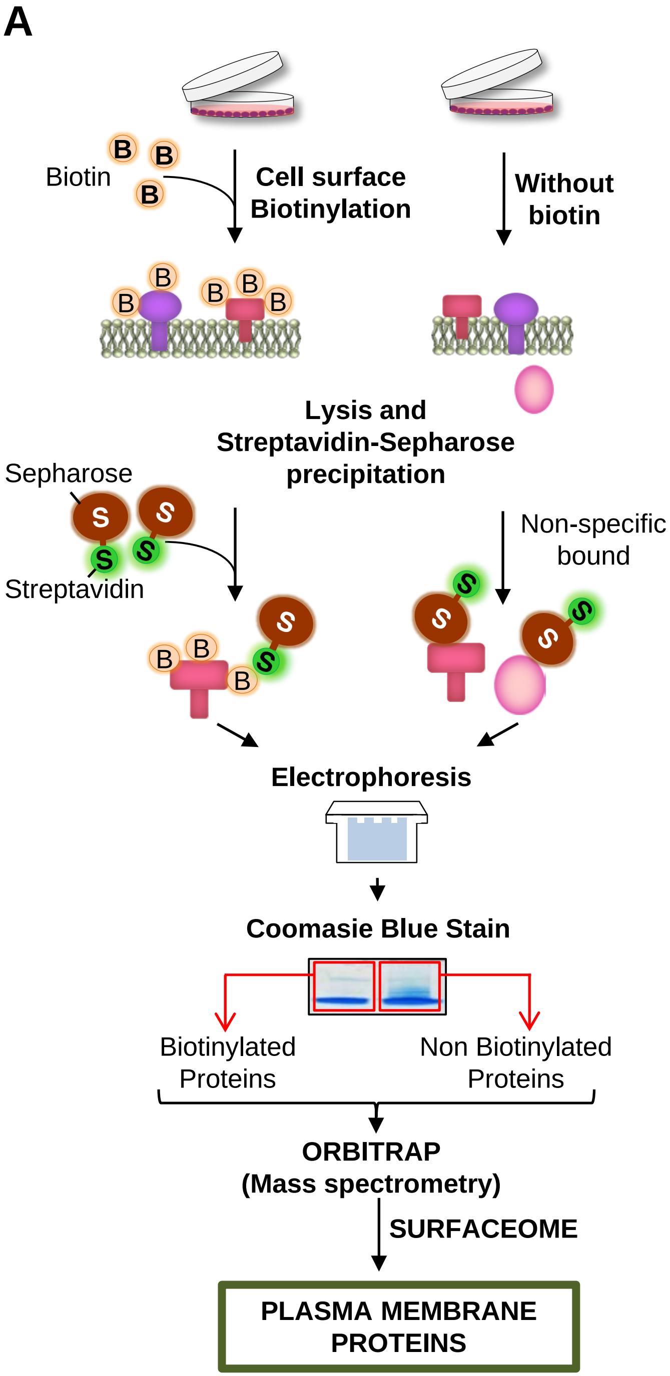

B

Cell line	Identified proteins	PLASMA MEMBRANE PROTEINS
MDA-MB-231	396	153
BT549	619	167
HS578T	990	229

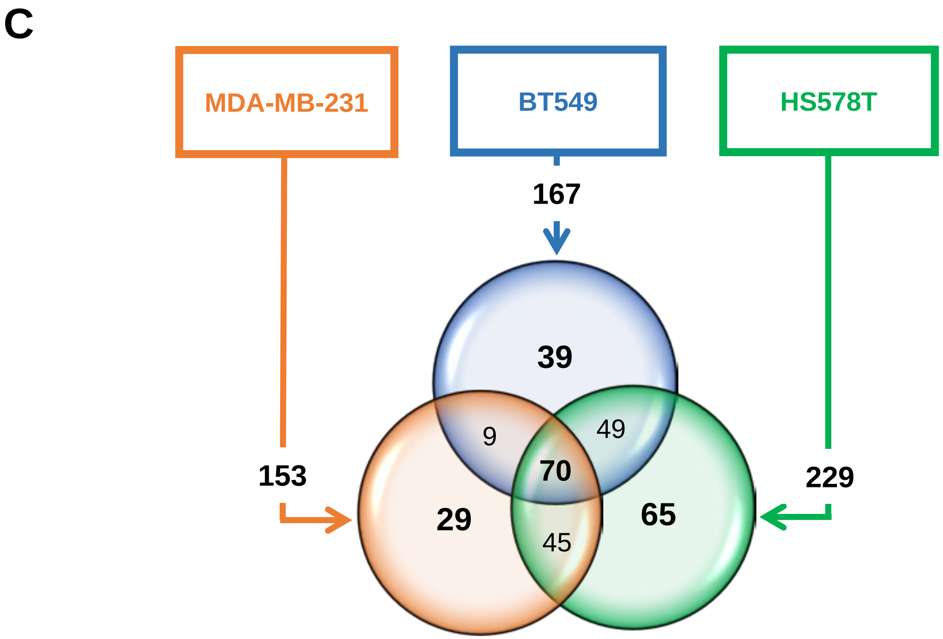

Supplementary Figure 4

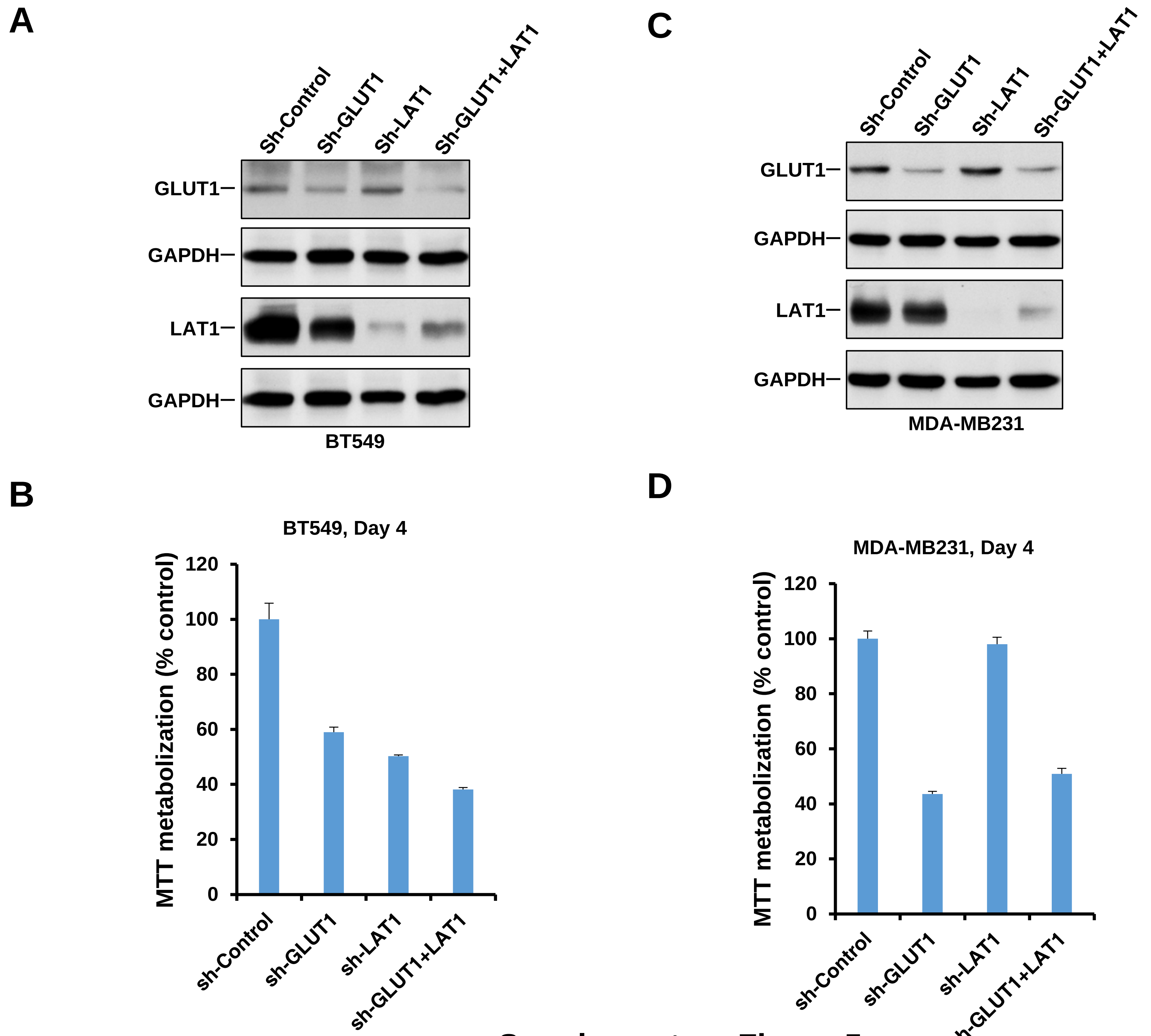

Supplementary Figure 5

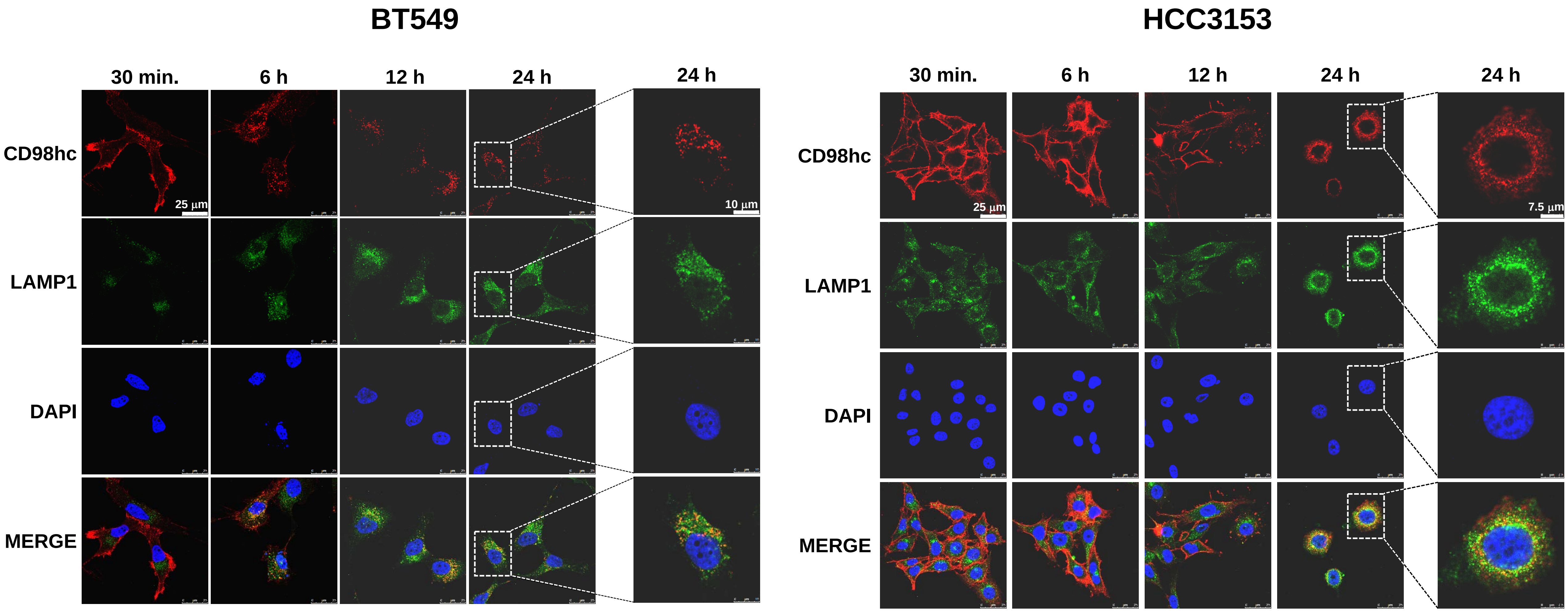

Supplementary Figure 6

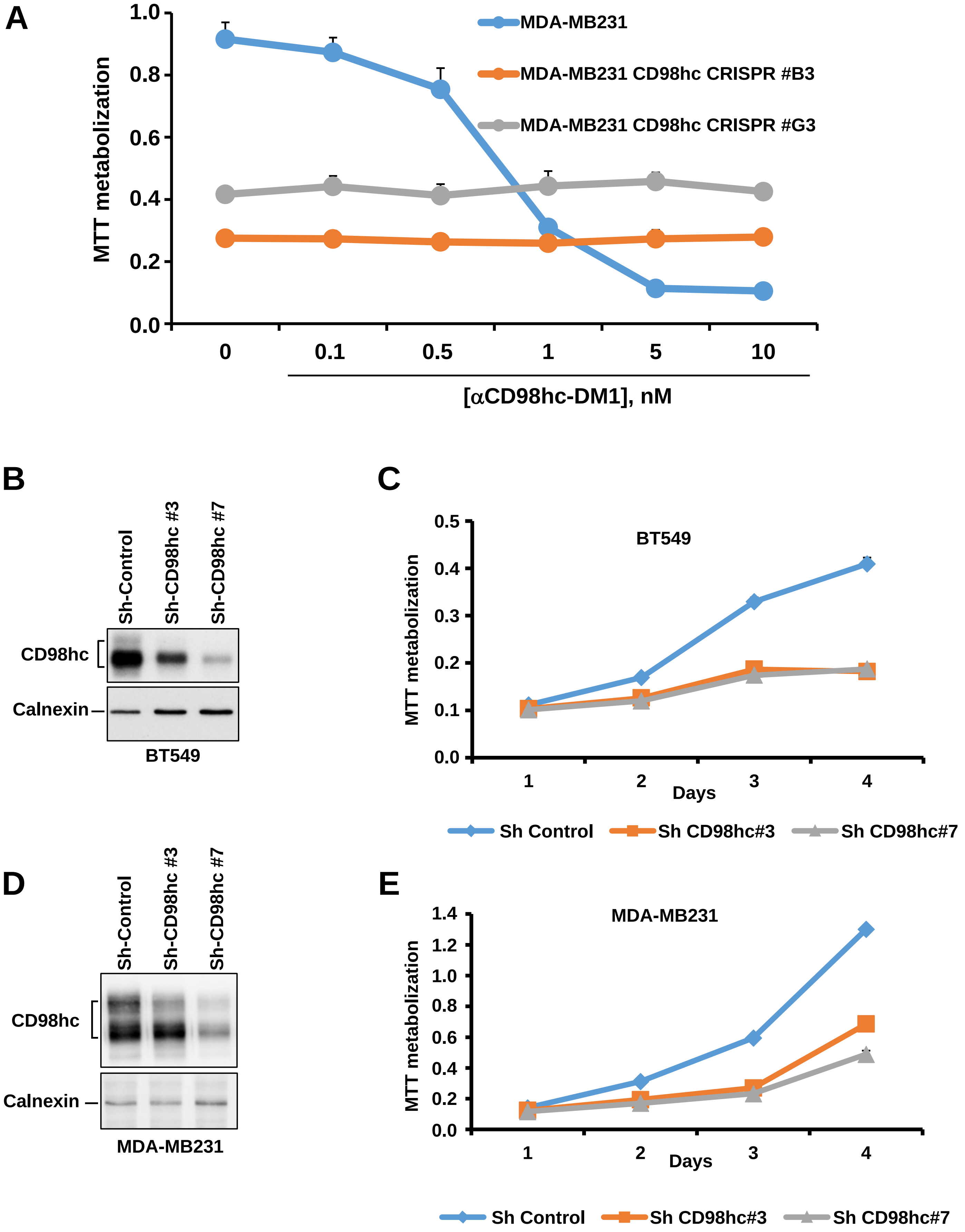

Supplementary Figure 7

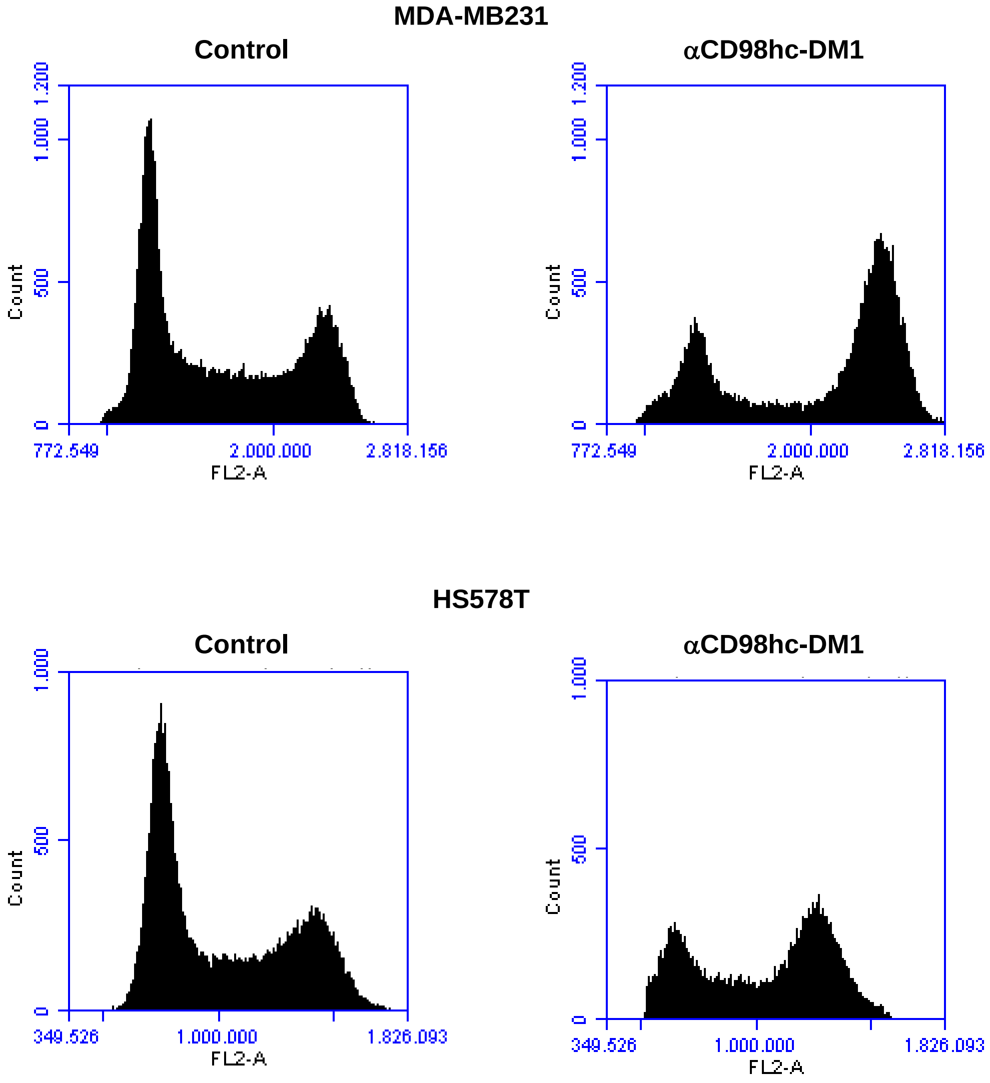

Supplementary Figure 8

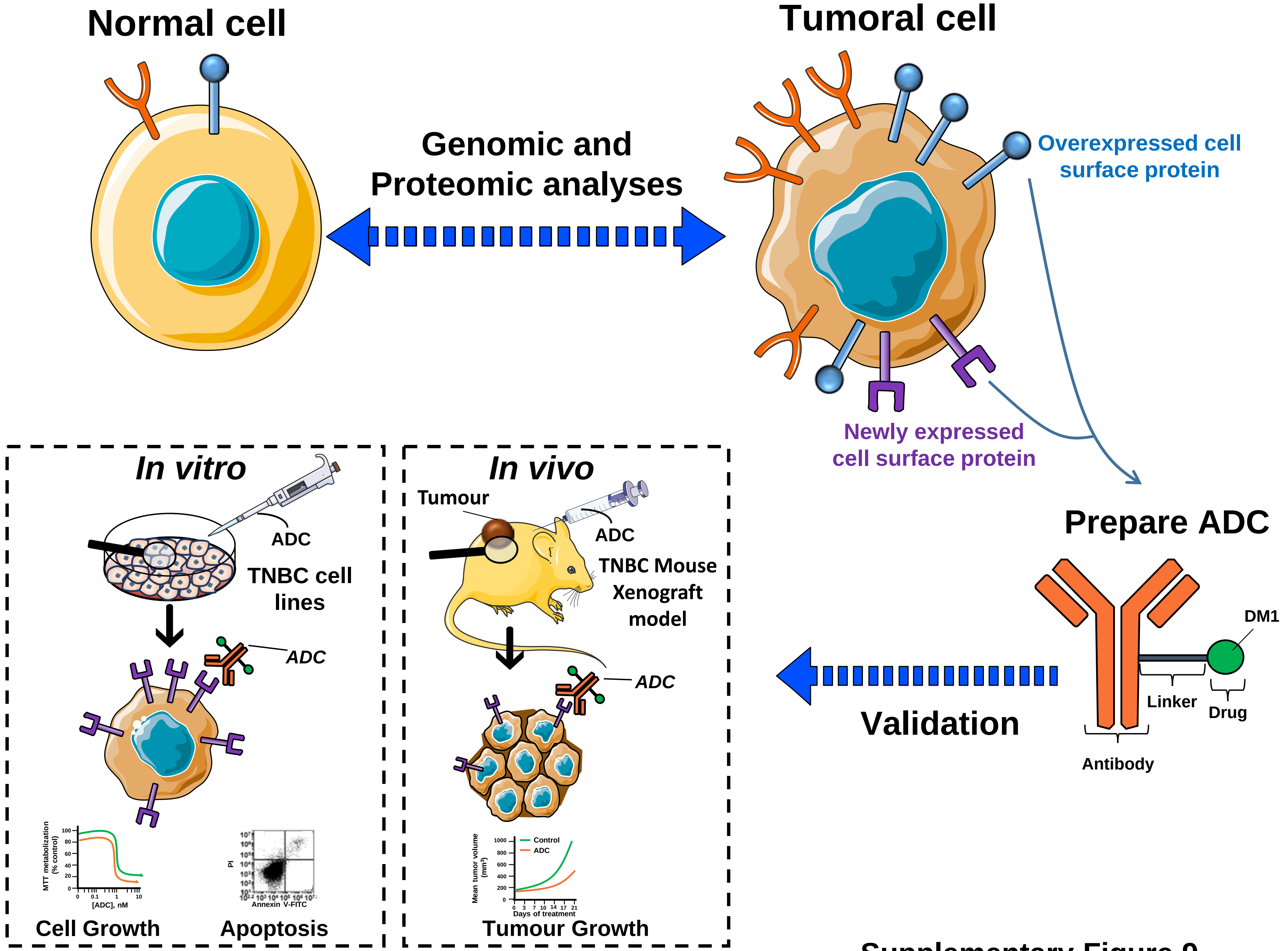

Supplementary Figure 9
